# Amentoflavone for treating cardiocerebrovascular diseases and neurological disorders

**DOI:** 10.3389/fphar.2024.1406510

**Published:** 2024-11-18

**Authors:** Hang Zhang, Yin-mei Ban, De-mei Li, Gang Wang, Juan Gu, Lei Zhu

**Affiliations:** ^1^ Department of Clinical Pharmacy, Key Laboratory of Basic Pharmacology of Guizhou Province and School of Pharmacy, Zunyi Medical University, Zunyi, Guizhou, China; ^2^ Key Laboratory of Basic Pharmacology of Ministry of Education and Joint International Research Laboratory of Ethnomedicine of Ministry of Education, Zunyi Medical University, Zunyi, Guizhou, China; ^3^ The Key Laboratory of Clinical Pharmacy of Zunyi City, Zunyi Medical University, Zunyi, Guizhou, China; ^4^ Department of Pharmacy, Affiliated Hospital of Zunyi Medical University, Zunyi, Guizhou, China

**Keywords:** amentoflavone, cardiovascular diseases, cerebrovascular diseases, neurological diseases, pharmacological effects, anti-inflammatory agents, Chinese herbal medicine

## Abstract

Amentoflavone (AME) is a flavonoid compound found in over 120 plants. Its extensive pharmacological activity for treating cardiocerebrovascular diseases and neurological disorders have attracted the attention of researchers in recent years. However, owing to the poor solubility and low bioavailability of AME, it has not been developed as a drug for treating these diseases. This review focuses on two aspects of AME: First, it provides a detailed summary and introduction to AME based on its chemical structure, physicochemical properties, plant sources, extraction and purification methods, administration systems, and pharmacokinetic properties. Second, it summarizes the effects of AME on cardiocerebrovascular diseases and neurological disorders, and its specific pharmacological mechanisms. This review aims to promote the use of AME for treating cardiocerebrovascular diseases and neurological disorders. AME exhibits multiple activities, indicating its potential as a natural drug for treating these diseases. Further studies on its pharmacokinetics and toxicology are required to ensure its safety and efficacy.

## 1 Introduction

Cardiocerebrovascular diseases refer to a group of conditions that affect the heart, brain, and other tissues. This category includes both cardiovascular and cerebrovascular diseases, as well as a variety of ischemic and hemorrhagic conditions. These diseases are precipitated by factors such as hyperlipidemia, thickened blood, atherosclerosis, and hypertension (Yan and Guo, 2022). Cardiovascular diseases are prevalent among individuals aged over 50 years, causing high morbidity and ranking first as a cause of death (Ma et al., 2021). In 2019, cardiovascular diseases accounted for approximately one-third of global deaths, with the highest number of deaths occurring in China (Roth et al., 2020). According to the 2022 *China Cardiovascular Health and Disease Report*, China currently has 13 million people with stroke, 11.39 million people with coronary heart disease, 8.9 million people with heart failure, 5 million people with cor pulmonale, 4.87 million people with atrial fibrillation, 2.5 million people with rheumatic heart disease, 2 million people with congenital heart disease, 45.3 million people with peripheral artery disease, and 245 million people with hypertension (The Writing Committee Of The Report On Cardivascular Health And Diseases and Hu, 2023). Neurological disorders are also widely prevalent. Neurological diseases were the second leading cause of death globally in 2015, resulting in approximately 9.4 million deaths, accounting for 16.8% of deaths. From 1990 to 2015, the number of deaths due to neurological diseases increased by 36.7% and disability-adjusted life years increased by 7.4% (Global Burden of Disease Study, 2013 Collaborators, 2015; Zhou et al., 2024). The high morbidity and mortality rates associated with these diseases urgently require effective treatment modalities to curb their progression (*Ginkgo biloba* L.). extract (GBE) has been approved for clinical use for treating various cardiovascular, metabolic, and neurodegenerative disorders (Peng et al., 2024). One of the critical components in GBE is flavonoids, which comprise up to 24% of its total content (Tao et al., 2022).

Amentoflavone (AME) is a flavonoid compound first isolated from the *Selaginella* plant (Okigawa et al., 1971). AME is one of the most common biflavonoid compounds found in *Ginkgo biloba* (Šamec et al., 2022). However, some studies suggest that the AME in *Ginkgo biloba* leaf extracts exhibits no biological activity and AME has been removed from the listing of the active components of such extracts (Xu et al., 2013). Recent research has demonstrated that AME has a wide range of pharmacological properties, including anti-inflammatory (Zhang and Wang, 2013), antioxidant (Li et al., 2020), anti-aging (Park and Kim, 2019), antibacterial (Bajpai et al., 2019), antiviral (Lee et al., 2023), anti-tumor (Qiu S. et al, 2021), antidepressant and anxiolytic (Ishola et al., 2012). AME also has beneficial effects on cardiocerebrovascular diseases and neurological diseases (Li et al., 2021; Saeedan et al., 2023; Sirimangkalakitti et al., 2019).

**FIGURE 1:**
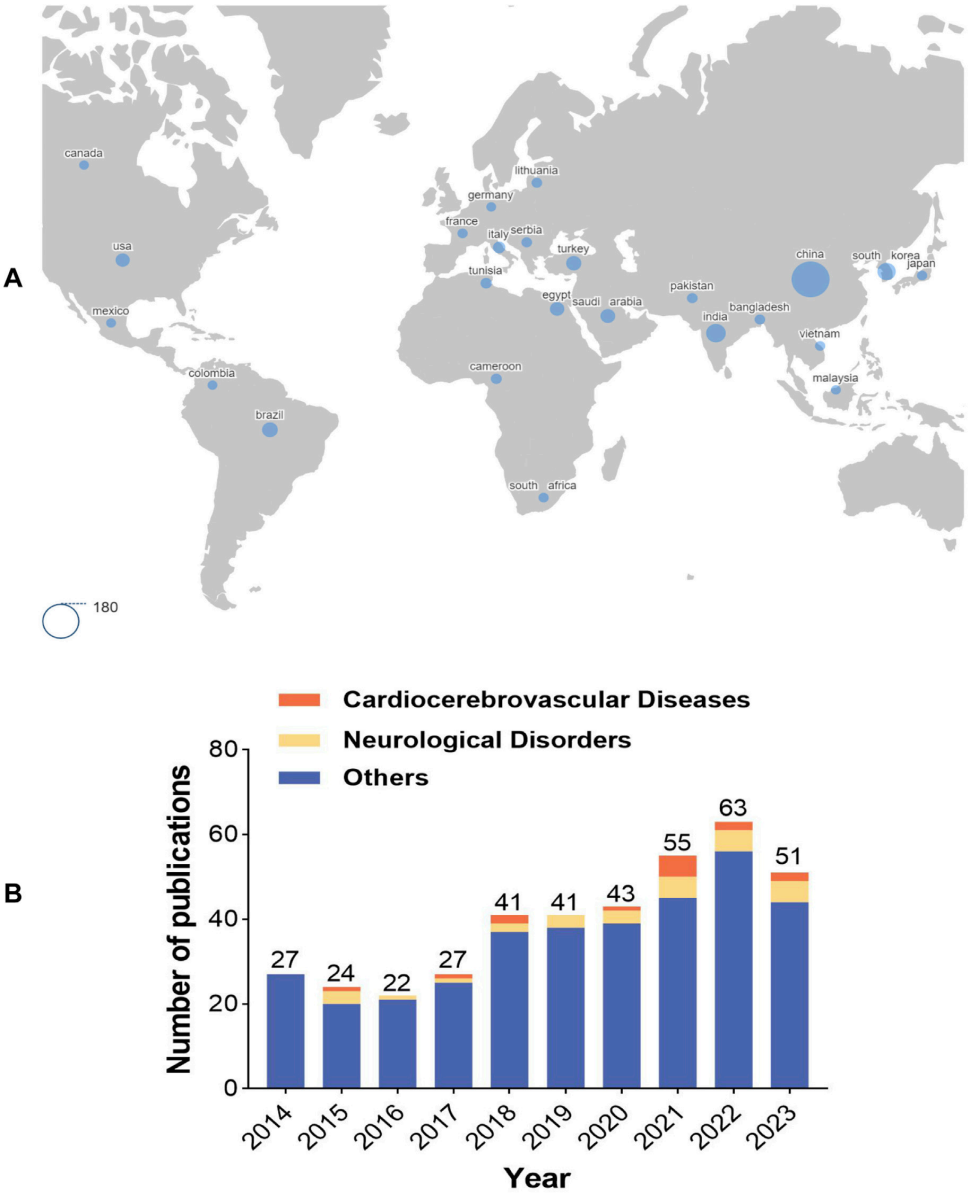
Bibliometric analysis of AME **(A)**. Global distribution map of countries involved in AME. The larger the blue circle in the figure is, the greater the number of articles published by the corresponding country. The area of the circle in the lower left corner is equivalent to 180 articles. **(B)**. Number of publications on AME in the past decade. Others represented other research fields of AME, with tumors and diabetes as the main research field.

A bibliometric analysis was conducted on the literature pertaining to AME from 2014 to 2023, providing insights into the research advancements in the field on a global scale. The keyword “Amentoflavone” was queried in the Web of Science database, yielding a total of 394 research papers from 63 countries and 641 institutions, authored by 2,196 individuals. Figure 1A displays the countries with the highest number of publications, providing insight into the level of interest in AME among these nations. Analysis of the data indicates a consistent upward trend in publication output over the past decade, with the number of articles published in recent years significantly surpassing those published in 2014. This trend suggests sustained interest and advancement in the field of AME, as depicted in Figure 1B. Research on the role of AME in cardiocerebrovascular diseases and neurological disorders has gained increasing attention since 2015, emerging as a current research hotspot in the field. Furthermore, the impact of AME on other conditions such as tumors and diabetes should not be underestimated.

This review provides a comprehensive summary of the chemical structure and physicochemical properties of AME, encompassing its botanical sources, extraction and purification techniques, and administration routes. Additionally, it examines the pharmacokinetic characteristics of AME, alongside its therapeutic effects on cardiocerebrovascular diseases and neurological disorders, elucidating the underlying pharmacological mechanisms. The objective of this review is to enhance the understanding of AME and its potential applications in the treatment of cardiocerebrovascular diseases and neurological disorders.

## 2 Background

### 2.1 Chemical structure and physicochemical properties of AME

**FIGURE 2:**
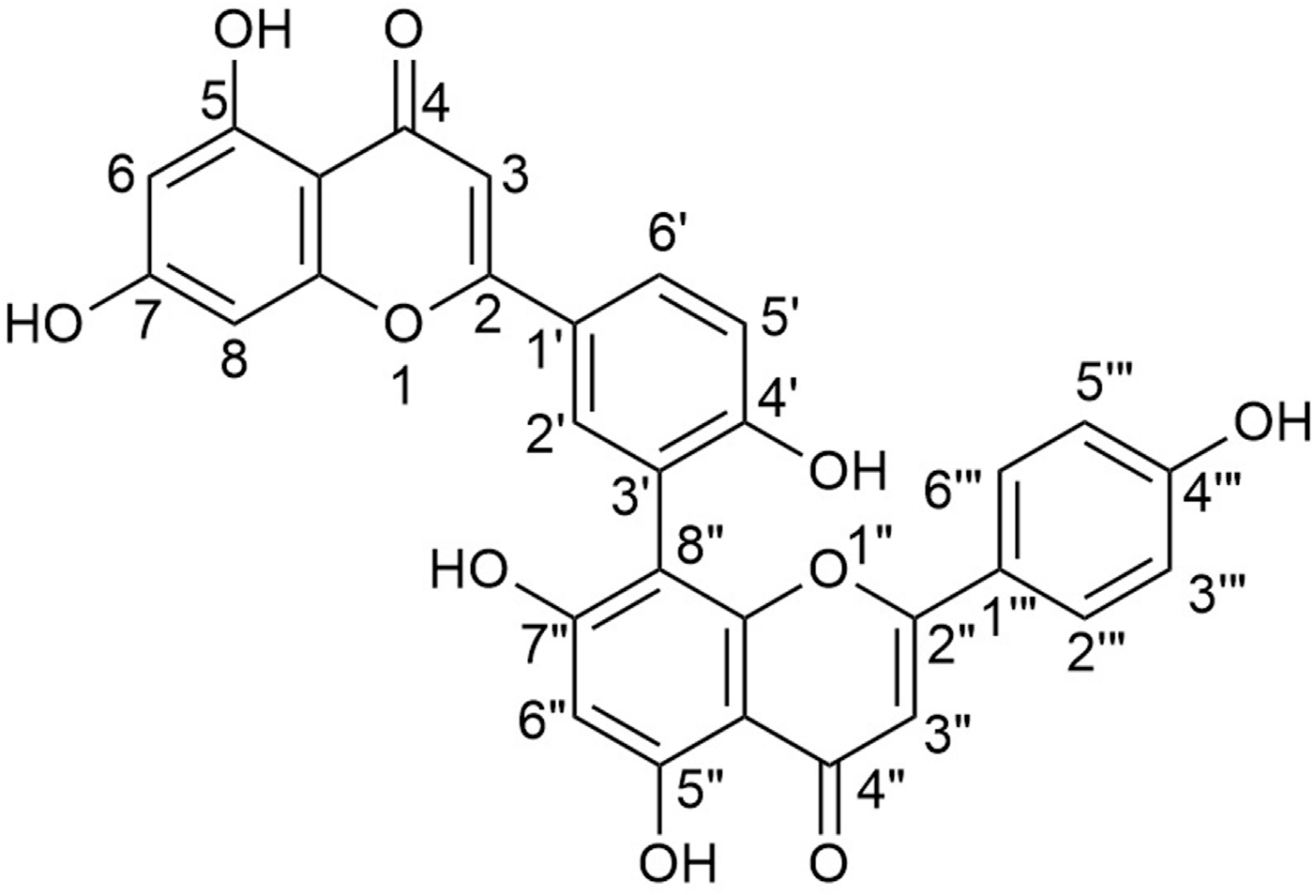
Chemical structure of amentoflavone (8-[5-(5,7-dihydroxy-4-oxo-4H-chromen-2-yl)-2-hydroxyphenyl]-5,7-dihydroxy-2-(4-hydroxyphenyl)-4H-chromen-4-one).

AME is a biflavonoid compound with the chemical name 8-[5-(5,7-dihydroxy-4-oxo-4H-chromen-2-yl)-2-hydroxyphenyl]-5,7-dihydroxy-2-(4-hydroxyphenyl)-4H-chromen-4-one. It is an apigenin dimer, with multiple double bonds and hydroxyl groups in its molecular structure (Figure 2). The C_2_-C_3_ double bond is susceptible to hydrogenation, whereas the hydroxyl group is prone to substitution with methoxy groups. Therefore, many hydroxylated derivatives have an amentoflavone nucleus (Yu et al., 2017; Šamec et al., 2022; Xiao et al., 2018). It has been reported that the hydroxyl groups at positions C_7_ and C_4'''_ are crucial for the anti-inflammatory activity of AME. Substitution of these hydroxyl groups with methoxy groups significantly reduces the anti-inflammatory efficacy (Mangmool et al., 2024). Additionally, the atropisomerism exhibited due to the C_3’_-C_8”_ linkage in the structure of AME may affect its binding to targets, potentially influencing its biochemical activities (Bhattacharya and Mandal, 2024).

The molecular formula of AME is C_30_H_18_O_10_, the molecular weight is 538.46 g/mol, and the melting point is 300°C (Xiong et al., 2021). The cross-conjugated molecular structure has strong characteristic absorption of ultraviolet light (Li, 2011). AME is a planar molecule with a close arrangement between molecules and large intermolecular attraction; therefore, it is poorly soluble in water, but is easily soluble in organic solvents such as ethanol and dimethyl sulfoxide (Xiong et al., 2021). Among the various polymorphs of AME, the amorphous form exhibits higher dissolution rates and solubility, and demonstrates good physical stability during the dissolution process (Zhou et al., 2022). It has been reported that the monomer apigenin from AME can interrupt free radical chain reactions and reduce the photoxidation process by generating resonance-stabilized free radicals (Huvaere et al., 2012). The presence of catechol structures and a double bond adjacent to the carbonyl group, along with hydroxyl groups on a single benzene ring, plays a crucial role in effectively quenching singlet oxygen (Nagai et al., 2005). This indirectly suggests that AME may exhibit strong antioxidant and photostability properties.

### 2.2 AME sources, and methods of extraction, separation, and purification

#### 2.2.1 Sources

AME is a bioactive compound present in numerous plant species. It was initially isolated from the leaves of (*Selaginella tamariscina* Maxim.), (*Selaginella rupestris* L.), and *Ginkgo biloba*. Subsequently, it has been extracted from over 120 plants species, including (*Celaenodendron mexicanum* L.), (*Cupressus funebris* Endl.), (*Garcinia multiflora* Bl.), and (*Hypericum perforatum* L.) (Xiong et al., 2021). The *Selaginella* genus, comprising 21 species, is the most prevalent source of amentoflavone. Other significant botanical families containing this compound include Cupressaceae, *Euphorbiaceae*, and *Clusiaceae*. Typically, AME is extracted from the leaves, aerial parts, and whole plants (Yu et al., 2017).

#### 2.2.2 Extraction methods

The principal methods for the extraction of AME include ultrasonic-assisted extraction, microwave-assisted extraction, organic solvent extraction, and semi-bionic extraction (Table 1). Each of these methodologies presents distinct advantages and disadvantages. For example, ultrasonic-assisted extraction offers benefits such as reduced extraction time, simplicity of operation, and high extraction efficiency. Nevertheless, its limited effective action area renders it unsuitable for industrial-scale production. Conversely, microwave-assisted extraction is noted for its straightforward operation, minimal byproduct formation, high extraction rates, and ease of product purification. However, it necessitates elevated extraction temperatures, which may compromise the integrity of active components. Organic solvent extraction is both cost-effective and straightforward; however, it suffers from low extraction efficiency, environmental pollution, and potential risks to human health make it less suitable (Li, 2011; Xu et al., 2021). Eutectic solvent (Liu et al., 2022) and infrared-assisted (Wang Y. et al., 2018) extraction techniques are gaining prominence in the extraction of AME due to their environmental sustainability, rapid processing times, and high efficiency.

**TABLE 1:** Methods of extracting amentoflavone.

Source	Extraction method	Extraction yield (%)	Reference
*Selaginella tamariscina*	Infrared-assisted extraction	0.290	Wang et al. (2018a)
*Selaginella tamariscina*	Ultrasonic-assisted ionic liquid extraction	1.351	Jiang et al. (2020)
*Selaginella tamariscina*	Ethanol reflux extraction	1.274	Wei (2010)
*Selaginella pulvinata*	Ethanol reflux extraction	1.728	Luo (2017)
*Selaginella sinensis*	Ionic liquid-microwave-based extraction	0.196	Li YY et al. (2019)
*Selaginella moellendorffii*	Deep eutectic solvent extraction	0.275	Liu et al. (2022)
*Selaginella doederleinii*	Ionic liquid-microwave-based extraction	0.650	Wang et al. (2018b)
*Selaginella doederleinii*	Microwave-assisted extraction	0.330	Wang et al. (2018c)
*Selaginella uncinata*	Ultrasonic-assisted extraction	1.530	Lai et al. (2018a)
*Podocarpus nagi*	Ethanol reflux extraction	0.010	Wang (2017)
*Cunninghamia lanceolata*	Ultrasonic-assisted extraction	0.319	Wang et al. (2023)
*Taxus chinensis*	Supercritical-CO_2_ fluid extraction	0.447	Ruan et al. (2014)

#### 2.2.3 Separation and purification methods

Currently, the primary methodologies for the separation and purification of AME are silica gel column chromatography, two-step precipitation, polyamide column chromatography, macroporous resin adsorption, and preparative high-performance liquid chromatography (HPLC) (Table 2). Researchers have been actively investigating enhanced techniques for separation and purification. Recently, flash chromatography has emerged as a novel method. Compared to traditional silica gel column chromatography, flash chromatography offers reduced the loading time, minimized dead adsorption, and improved efficiency and product purity. However, it exhibits lower separation capacity and is prone to interference from metal ions during elution. Macroporous resin adsorption offers several advantages, including rapid high-capacity and selective adsorption, as well as high elution efficiency, rendering it suitable for large-scale production. Nonetheless, its desorption efficiency remains suboptimal, and the purification rate is affected by the type of eluent and temperature (Xu et al., 2021). HPLC offers advantages including wide applicability, exceptional quantitative capabilities, and well-recognized methodologies. Nevertheless, it is marked by the necessity for extensive sample preparation, prolonged analysis durations, and expensive instrumentation (Vlasiou, 2023).

**TABLE 2:** Methods of separating and purifying amentoflavone.

Source	Separation and purification method	Purity (%)	Recovery (%)	Reference
*Selaginella tamariscina*	Two-step precipitation method	58.2	88.7	Wei (2010)
*Selaginella tamariscina*	High-speed countercurrent chromatography	99.2	94.7	Wei (2010)
*Selaginella tamariscina*	Silica gel column chromatography	97.2	50.7	Wei (2010)
*Selaginella tamariscina*	Low pressure column chromatography	98.7	87.8	Wei (2010)
*Selaginella pulvinata*	Macroporous adsorption resin (HPD 300)	52.5	62.4	Luo (2017)
*Selaginella pulvinata*	Two-step precipitation method	47.9	59.7	Luo (2017)
*Selaginella uncinata*	Macroporous adsorption resin (NKA-9)	N/A	64.3	Lai et al. (2018b)
*Selaginella moellendorffii*	Macroporous adsorption resin (D-101)	80.8	62.5	Fang et al. (2011)
*Podocarpus nagi*	Macroporous adsorption resin (AB-8)	93.6	N/A	Wang (2017)

### 2.3 AME dosing forms

AME exhibits significant pharmaceutical potential and promise as a therapeutic agent (Xiong et al., 2021; Li et al., 2021). However, its poor water solubility constrains its release within the body, resulting in incomplete gastrointestinal absorption and low oral bioavailability, thereby limiting its pharmacological efficacy (Ren et al., 2013a). Following oral administration in rats, AME is dominantly distributed in the small intestine, stomach, liver, and large intestine, with minimal distribution to other tissues. To maximize the pharmacological efficacy of AME, it is imperative to enhance its solubility and bioavailability. Key strategies for improving drug solubility and bioavailability include modifying the delivery method, formulation (Table 3), and structural enhancement (Feng et al., 2020).

**TABLE 3:** Different formulations to improve amentoflavone.

Route of administration	Formulation	Particle size	Zeta potential	Improved results	Reference
Oral	Micro-emulsion formulation	15.37 ± 0.09 nm	−17.1 ± 0.24mV	Dissolution rate	Ren et al. (2013a)
Oral	Micro-powder formulation	0.08 ± 0.01 μm	N/A	Solubility, dissolution rate	Ren et al. (2013b)
Intravenous	Nanoparticulate formulation	77.3 ± 5.3 nm	−2.92 ± 0.27 mV	Solubility, dispersibility, stability, bioavailability, reduce toxicity	Zhao et al. (2022)
Oral	Micelle formulation	58.8 ± 1.29 nm	5.26 ± 0.63 mV	Bioavailability	Zhang et al. (2019)
Oral	Micelle formulation	67.33 ± 2.01 nm	−0.84 ± 0.04 mV	Bioavailability	Feng et al. (2020)
Oral	Sub-micron particle formulation	Approximately 0.4 μm	N/A	Solubility, dispersibility, stability, dissolution rate	Duan et al. (2022)
Oral	Micelle formulation	25.99 ± 0.10 nm	−27.67 ± 0.25 mV	Solubility, dissolution rate; Bioavailability	Feng et al. (2023)
Intranasal	Nanoemulsion formulation	Approximately 37 nm	Approximately −4 mV	Bioavailability	Khafagy et al. (2023)

### 2.4 Pharmacokinetics of AME

Despite the multiple beneficial biological properties of AME, its pharmacokinetics remain inadequately characterized. A comprehensive understanding of AME’s pharmacokinetics is crucial for elucidating its *in vivo* mechanisms of action, characterizing its properties, and optimizing drug design and dosing, to maximize therapeutic efficacy. Insights into the fundamental pharmacokinetics of AME can be inferred from studies conducted in animal models (Table 4).

**TABLE 4:** Pharmacokinetic parameters of amentoflavone.

Route of administration	Dose	*C*_max_	*T*_max_	*T*_*1/2*_	AUC_0-t_	AUC_0-∞_	*CL/F*	*V/F*	MRT_0-t_	MRT_0-∞_	*F* (%)	Reference
Oral	2.8 g/kg	22.5 ± 1.4 ng/mL	1.13 ± 0.44 h	2.06 ± 0.13 h	125 ± 7 ng/h mL	133 ± 8 ng/mL h	N/A	N/A	N/A	N/A	N/A	Wang et al. (2014)
Intragastric	60 mg/kg	0.469 ± 0.046 min/L	90.0 min	57.01 ± 2.765 min	27.7 ± 1.1 mg/L min	24.9 ± 1.1 mg/L min	2.4 ± 0.1 L min^−1^ kg^−1^	198.4 ± 17.4 L/kg	122.8 ± 1.6 min	126.1 ± 2.3 min	N/A	Wang et al. (2015)
Oral	500 mg/kg	42.37 ± 11.95 ng/mL	0.85 ± 0.137 h	12.33 ± 4.65 h	194.5 ± 16.9 ng/mL h	299.2 ± 75.4 ng/mL h	N/A	N/A	9.58 ± 0.84 h	N/A	0.06 ± 0.04	Gan et al. (2020)
Intravenous	10 mg/kg	17,505 ± 1,532 ng/mL	0.033 h	9.36 ± 2.97 h	10,060.9 ± 1,163.8 ng/mL h	10,706.6 ± 1,225.9 ng/mL h	N/A	N/A	3.365 ± 0.34 h	N/A	N/A	Gan et al. (2020)
Intravenous	10 mg/kg	31.2 ± 27.0 nmol/mL	0.11 ± 0.02 h	5.88 ± 1.78 h	33.0 ± 11.9 nmol/mL h	35.2 ± 13.9 nmol/mL h	320 ± 139 mL h^−1^ kg^−1^	2.53 ± 0.65 L/kg	N/A	N/A	N/A	Liao et al. (2015)
Intraperitoneal	10 mg/kg	6.26 ± 0.33 nmol/mL	0.83 ± 0.29 h	3.42 ± 1.45 h	25.5 ± 1.08 nmol/mL h	25.7 ± 0.82 nmol/mL h	N/A	N/A	N/A	N/A	77.4 ± 28.0	Liao et al. (2015)
Oral	300 mg/kg	0.06 ± 0.03 nmol/mL	0.33 ± 0.14 h	11.3 ± 3.6 h	0.41 ± 0.08 nmol/mL h	0.49 ± 0.12 nmol/mL h	N/A	N/A	N/A	N/A	0.04 ± 0.01	Liao et al. (2015)
Oral	4.31 mg/kg	124.61 ± 8.37 ng/mL	1.5 h	2.60 ± 1.34 h	594.48 ± 62.12 μg h/L	597.84 ± 60.41 μg h/L	7.27 ± 0.75 L min^−1^ kg^−1^	N/A	N/A	N/A	N/A	Shan et al. (2018)

*C*_max_, maximum blood concentration; *T*_max_, time to peak concentration; *T*_*1/2*_, biological half-life; AUC(0-t), area under the concentration-time cure; AUC(0-∞), from time zero to all original drug elimination; *CL/F*, clearance; *V/F*, apparent volume of distribution; MRT_0-∞_, mean residence time; MRT_0-t_, average retention time for a certain period of time; *F*, bioavailability.

It has been demonstrated that AME is absorbed via passive diffusion in rats (Wei et al., 2017). Furthermore, a study employing the Caco-2 cell model indicated that AME exhibits intestinal absorption. The absorption may involve paracellular passive diffusion and clathrin-mediated endocytosis, while the efflux transporter appears to be uninvolved (Wang B. et al., 2020). The low bioavailability of AME is due to extensive glucuronidation catalyzed by uridine diphosphate glucuronosyltransferase 1 family, polypeptide A (UGT1A1) and UGT1A3 (Gan et al., 2020).

Another study conducted demonstrated that the peak blood concentration in rats was reached at 90 min following oral administration, with a volume of distribution (V/F) of 198.36 ± 17.422 L/kg. These findings suggest that AME is predominant distributed or sequestered in specific tissues and organs within the rat model. (Wang et al., 2015).

*In vivo*, 34 metabolites of AME were identified, while 24 metabolites were identified *in vitro*. In the *in vivo* study, all metabolites were distributed in feces, with three of the 34 metabolites were also detected in urine, and none were found in bile or plasma. In the *in vitro* study, 20 of the 24 metabolites were distributed in liver microsomes, and 17 of the 24 metabolites were associated with the intestinal microbiota. Of the total metabolites identified both *in vivo* and *in vitro*, 14 were classified as phase I metabolites and 26 as phase II metabolites. The primary metabolic pathways included oxidation, methylation, acetylation, and oxidation methylation (Feng, 2020). It has been reported that the bioactive form of AME is likely conjugated (Liao et al., 2015). Another study found that 90.7% of AME circulates as conjugated metabolites post-administration. In rats, 73.2% and 70.2% of AME in plasma were in conjugated form following intravenous and intraperitoneal injection, respectively (Yu et al., 2017).

The cumulative excretion rates of AME in feces and urine were 23.93% and 0.82%, respectively, indicating that fecal excretion is the predominant route of AME elimination from the body. This high rate of fecal excretion may contribute to the compound’s low bioavailability (Chen et al., 2022).

## 3 Biological activity of AME for treating cardiocerebrovascular and neurological diseases

**FIGURE 3:**
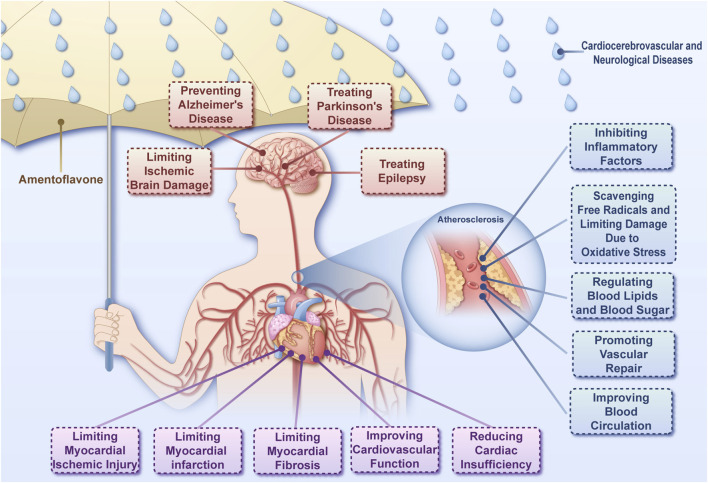
The ameliorative effect of amentoflavone on cardiocerebrovascular and neurological diseases Amentoflavone is capable of acting on cardiocerebrovascular diseases, neurological diseases through a variety of mechanisms.

Atherosclerosis is a main pathological basis for cardiovascular and cerebrovascular diseases (Dutta et al., 2023). The cerebrovascular system is closely related to the structure and function of brain tissue. Vascular factors are important in the development of neurological diseases (Iadecola, 2023; Smith et al., 2021). Therefore, this section discusses the effect of AME on cardiocerebrovascular and neurological diseases (Figure 3).

### 3.1 Potential mechanisms of AME in atherosclerosis

**FIGURE 4:**
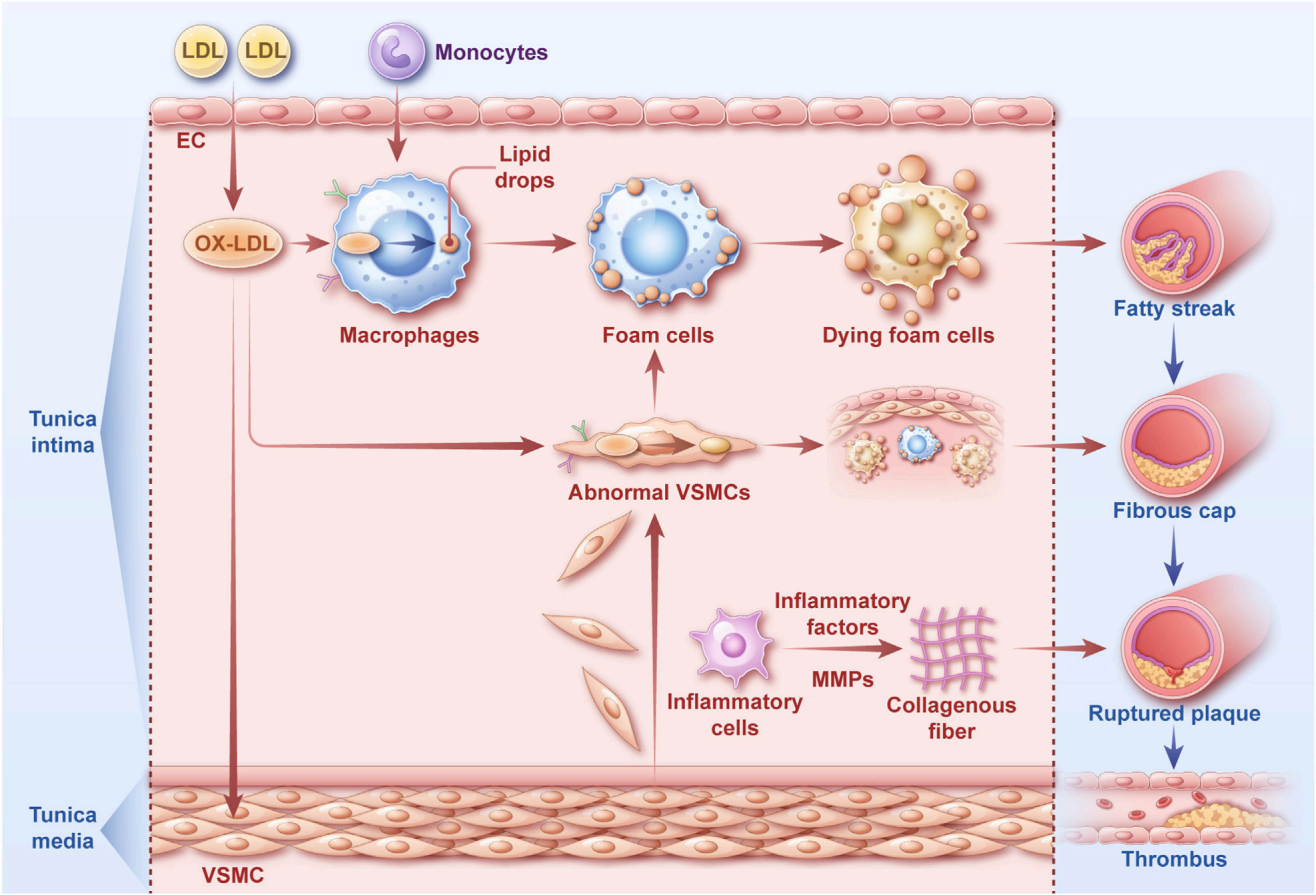
The atherosclerotic process The key steps of the atherosclerotic process can be divided into the following steps: Formation of oxidized low-density lipoprotein and activation of macrophages; Macrophage-derived foam cell formation; Abnormal VSMCs and myogenic foam cell formation; Formation and rupture of atherosclerotic plaque.

Although there is currently no published literature regarding the use of AME in the treatment of atherosclerosis, its various potential mechanisms of action are promising. The process of atherosclerotic plaque formation can be divided into the following steps (Figure 4): (1) When the vascular wall is damaged, low-density lipoprotein (LDL) enters the intimal layer of the blood vessel through the gaps between endothelial cells (ECs), forming oxidized low-density lipoprotein (ox-LDL). Monocytes migrate into the intimal layer and are activated into macrophages. (2) Ox-LDL binds to receptors on the surface of macrophages, and cholesterol enters the macrophages, where it is esterified. When the intake, esterification, and release of cholesterol are out of equilibrium, intracellular lipid overload leads to the formation of macrophage-derived foam cells, creating fatty streaks in the lesions (Gui et al., 2022). (3) Ox-LDL induces changes in the phenotype of vascular smooth muscle cells (VSMCs) in the tunica media of the arterial wall, leading to abnormal proliferation and migration to the intimal layer. Subsequently, VSMCs engulf ox-LDL to form myogenic foam cells (Yang, 2023; He, 2013), which then form a fibrous plaque. (4) Necrosis and disintegration of macrophage-derived and myogenic foam cells lead to atherosclerotic plaque formation. Inflammatory cells secrete matrix metalloproteinases (MMPs) to break down collagen fibers in the extracellular matrix, thereby increasing plaque instability. Subsequently, the plaque ruptures, causing bleeding and thrombosis (Carracedo et al., 2019; Libby, 2021; Poznyak et al., 2020). Atherosclerosis is a chronic inflammatory disease involving various inflammatory, free radical, and oxidative stress-related injuries (Meng et al., 2024).

#### 3.1.1 Inhibiting inflammatory factors

When inflammation occurs *in vivo*, IκB kinase phosphorylates inhibitor of NF-κB (IκB) protein, and then IκB protein is separated from the p50 and p65 subunits of nuclear factor-kappa B (NF-κB), so that NF-κB is activated and enters the nucleus for gene transcription and expression of inflammatory factors, inflammatory mediators, chemokines, and adhesion factors (Guo et al., 2024). AME can protect vascular ECs through various mechanisms. AME increases the survival of human umbilical vein endothelial cells (HUVECs) induced by TNF-α in the S phase of cell proliferation. Disruption of the endothelin-1 (ET-1)/nitric oxide (NO) balance in the blood is an indicator of vascular endothelial damage. AME can increase the NO content of HUVECs, reduce the level of ET-1, inhibit the expression of adhesion factors vascular cell adhesion molecule-1 and E-selectin, and inflammatory factors interleukin IL-6 and IL-8, enhance the expression of the inhibitory protein IκBα activated by NF-κB, and reduce the expression of its transcription factor NF-κB in the nucleus, preventing further damage to the vascular endothelium (Zheng et al., 2013). AME downregulates the release of NO from mouse macrophages stimulated by lipopolysaccharide (LPS), and the levels of TNF-α and IL-1β induced by LPS and monosodium urate in THP-1 macrophages, via the NOD-like receptor thermal protein domain associated protein 3 (NLRP3)/apoptosis-associated speck-like protein/cysteinyl aspartate specific protease (caspase)-1 signaling pathway (Zhang X. et al., 2021). An experiment was conducted to examine the binding effects of 34 flavonoid products on the NLRP3 inflammasome, using CB-Dock molecular docking for binding predictions. It was found that AME has the strongest affinity for the NLRP3 inflammasome, surpassing that of its specific inhibitor (CY-09) (Fang H. Y. et al., 2023). Macrophage migration inhibitory factor (MIF) is a crucial pro-inflammatory mediator. AME has been reported to exhibit superior inhibitory activity against MIF compared to ISO-1, a well-established standard MIF inhibitor (Siddiqui et al., 2024).

During inflammation, arachidonic acid is converted to prostaglandin E−2 (PGE-2) by cyclooxygenase-2 (COX-2) through the action of cyclooxygenase-2, leading to pain and inflammation. At a concentration of 50 μM, AME can inhibit the activity and expression of COX-2 induced by TNF-α in cells, and upregulate the activity of peroxisome proliferator-activated receptor (PPAR) γ, blocking the degradation of IκBα, and inhibiting the translocation of NF-κB to inhibit the activation of the NF-κB signaling pathway (Banerjee et al., 2002). AME isolated from the root of (*Prismatomeris glabra* Mart.) exerts an anti-inflammatory effect by significantly reducing the production of TNF-α, IL-6, and PGE-2 in THP-1-derived macrophages in a dose-dependent manner (Alkadi et al., 2021). AME effectively suppresses the production of NO and PGE-2 in RAW264.7 macrophage cells stimulated by LPS. This inhibitory effect is achieved by inhibiting the kinase activity of extracellular signal-regulated kinase (ERK). Furthermore, AME also inhibits the expression of inflammation-related genes induced by LPS, including nitric oxide synthase (iNOS), COX-2, and TNF-α (Oh et al., 2013).

Macrophages of different polarization types are involved in distinct atherosclerosis development processes. Specifically, M1-type macrophages are primarily present in early plaques, associated with plaque formation. M2-type macrophages can exert anti-inflammatory effects, promoting the repair of atherosclerosis inflammation. PPARs are a class of ligand-activated transcription factors that play a crucial role in macrophage polarization, regulating macrophage metabolism, suppressing pro-inflammatory genes, and promoting the transformation of M2 macrophage phenotype (Zhuang, 2020). PPARγ, through its interaction with other transcription factors and the promoter regions of arginase 1 (*Arg1*), found in inflammatory zone (*Fizz1*), and chitinase-like protein 3 (*Ym1*) genes, promotes the expression of *Arg1* and *Fizz1* genes and regulates the M2 polarization level of macrophages. Macrophages lacking PPARγ exhibit significantly downregulated expression of *Arg1*. AME can inhibit the differentiation of THP-1-derived M0 cells toward M1 cells by activating PPAR-α/γ transcription factors, elevating the mRNA levels of TGF-β and IL-10, reducing TNF-α and IL-6 expression, and upregulating *Arg1* and *Fizz1* protein expression (Qiu F. et al., 2021). These findings suggest that AME might also reverse the transformation of M1 macrophages toward the M2 type, thereby exerting an anti-inflammatory effect.

#### 3.1.2 Scavenging free radicals and limiting damage due to oxidative stress

NO produced iNOS in activated macrophages is one of the most important inflammatory mediators. iNOS-mediated NO production and the associated production of highly reactive free radicals such as peroxynitrite has a harmful effect (Wang Y. et al., 2020). Therefore, inhibiting NO may be a useful target for addressing oxidative stress.

*In vitro* antioxidant models have demonstrated that AME exhibits exceptional scavenging and antioxidant capabilities when eliminating (1,1-diphenyl-2 trinitrophenylhydrazine) DPPH free radicals, superoxide anions, and hydroxyl radicals. Additionally, AME possesses the ability to repair and protect DNA from oxidative damage *in vitro*. AME influences the generation and scavenging of OH free radicals, and eliminates free radicals by scavenging hydrogen ion and electron. Therefore AME can treat oxidative damage (Wang, 2013). AME can eliminate DPPH free radicals in a concentration-dependent manner and can alleviate the cell damage caused by ox-LDL in HUVECs. The mechanism might be due to the phenolic hydroxyl group in the molecular structure of AME, which can accept the electron transfer from lipid peroxidation free radicals, forming stable free radicals, thus preventing the damage of lipid peroxidation free radicals to vascular ECs (Xu et al., 2004). Advanced glycation end-products (AGEs) are associated with various diseases such as atherosclerosis and diabetes, leading to the production of reactive oxygen species (ROS), which subsequently activates the transcription factor NF-κB and is involved in various inflammatory diseases. Ferchichi et al. extracted various active components from plants, and showed that AME had the strongest *in vitro* ability to effectively resist AGEs and exhibits excellent scavenging capabilities (Ferchichi et al., 2012). Furthermore, AME can block the nuclear translocation of NF-κB p65, inhibit the phosphorylation of IκBα and formation of NO in macrophages induced by LPS in a concentration-dependent manner by blocking the degradation of IκBα to inhibit the formation and transcription activation of the iNOS gene induced by LPS (Woo et al., 2005).

AME reduces LPS-induced oxidative stress damage to HUVECs, increasing the activity of superoxide dismutase (SOD) and reducing the expression levels of NO and malondialdehyde (MDA). A multi-omics study found that glycine, argininosuccinic acid, putrescine, ornithine, spermidine, 5-oxoproline, and dihydrouracil are seven metabolites that might be related to the mechanism by which AME protects ECs (Yao et al., 2016).

The reduced form of thioredoxin (Trx) interacts with the N-terminus of apoptosis signal regulating kinase 1 (ASK1) both *in vitro* and *in vivo*, thereby inhibiting ASK1 activity. Under conditions of oxidative stress, the thiol group of the cysteine residue of Trx is oxidized to form intramolecular disulfide bonds, thereby activating ASK1 kinase activity. Therefore, in the complex of Trx with ASK1 protein, Trx1 and thioredoxin reductase (TrxR)-1 proteins are the key molecules regulating ROS-induced ASK1 activation. AME can regulate the ROS/ASK1/p38 mitogen-activated protein kinase (MAPK) pathway by inactivating ASK1 molecules, blocking p38 MAPK signaling, increasing the levels of thioredoxin Trx1 and reductase TrxR-1, and reducing ASK1 and p38 MAPK levels, thereby reducing oxidative stress damage to cells (Li et al., 2020). These studies demonstrate that AME can respond to oxidative stress through various mechanisms.

#### 3.1.3 Regulating blood lipids and blood sugar

The fat accumulation index can serve as a predictive indicator of cardiometabolic diseases and stroke. The fat accumulation index is linearly related to the risk of atherosclerotic cardiovascular disease within 10 years and is an independent risk factor, indicating an integral relationship between body fat and atherosclerotic diseases (Liu et al., 2023). A randomized trial involving over 20 million participants demonstrated that blood lipid components such as LDL, apolipoprotein A, apolipoprotein B, and triacylglycerols can also affect the development of atherosclerosis (Ference et al., 2017). Therefore, controlling the level of blood lipids can alleviate and prevent atherosclerosis.

Obesity leads to the storage of excess energy in the form of triglycerides (TG) in adipose tissue. Dietary fat can lead to increased TG levels in the blood and obesity, whereas lipid absorption disorders can lead to hyperlipidemia and metabolic diseases. The cluster of differentiation 36 (CD36) protein primarily participates in the process of macrophage lipid uptake. When a large amount of ox-LDL appears in the vascular endothelial layer, the CD36 protein is activated to take up ox-LDL into the cell. After intake, ox-LDL is oxidized to new derivatives by linoleic acid, and these new derivatives can activate PPARγ, thereby promoting the increase in PPARγ protein expression as a transcription factor of CD36. Increased CD36 expression in turn promotes ox-LDL uptake. AME can reduce the uptake of ox-LDL by THP-1-derived macrophages through the CD36/PPARγ signaling pathway, thereby inhibiting foam formation induced by ox-LDL (Zhuang, 2020). Feeding AME to mice on a high-fat diet reduced the expression of the lipid absorption-related gene, CD36, by affecting plasma TG levels, thereby affecting the intestinal absorption of lipids (Lee et al., 2022). AME can reduce the body weight, total fat tissue, and serum TG content induced by a high-fat diet in a dose-dependent manner. AME has been demonstrated to reduce blood glucose levels and insulin resistance in rat models. Furthermore, AME influences various stages of 3T3-L1 adipocyte differentiation. Specifically, it modulates ROS production and inhibits the expression of the transcription factor CCAAT/enhancer-binding protein (C/EBP)-β during the mitotic clonal expansion (MCE) phase, thereby impacting MCE. Additionally, AME downregulates the expression of PPARγ and C/EBP-α during both the early and terminal differentiation phases, consequently affecting lipid droplet formation. (Chen et al., 2016).

Type 2 diabetes is intricately linked to atherosclerosis. Patients with diabetes often have disorders of lipid metabolism and insulin resistance syndrome. Elevation of blood glucose and lipid levels in patients with type 2 diabetes are risk factors for atherosclerosis, and are positively correlated with the extent of atherosclerosis lesions (Sun et al., 2023; Huang and Sun, 2023). High blood glucose levels disrupt ECs function, causing damage the vascular wall, thereby promoting atherosclerosis progression (Wei and Liang, 2021). AME significantly suppresses the elevation of blood glucose levels and reduces the Homeostatic Model Assessment of Insulin Resistance index. Furthermore, they demonstrated that AME also reduced lipid accumulation in the liver of high fructose and fat diet (HFFD)-fed rats and alleviated the damage caused by lipids to the liver (Qin et al., 2018). AME reduced the expression of genes related to fat production and upregulated the expression of genes related to insulin signaling transmission, thereby having anti-obesity and anti-hyperglycemic effects (Cho et al., 2021).

*In vitro* hypoglycemic effects of AME using HepG2 cell glucose models and insulin resistance models and found that AME significantly increased glucose metabolism of HepG2 cells and had a synergistic effect on the increased glucose consumption under insulin stimulation. In the insulin resistance HepG2 cell model induced by high insulin, AME also increased the glucose consumption of cells that developed insulin resistance (Zheng et al., 2008). These characteristics suggest that AME may serve as an insulin sensitizer, increasing cell glucose consumption and synergistically working with insulin to reduce blood glucose levels and reduces insulin resistance. In the insulin resistance HepG2 cell model induced by high glucose and high insulin, AME significantly increased the levels of rate-limiting enzymes for glucose oxidative decomposition, such as 6-phosphogluconate kinase, glucose kinase, and pyruvate kinase; reduced glucose synthesis by lowering glucose synthesis kinase-3-β (GSK-3-β) levels; and lowered phosphoenolpyruvate carboxy kinase and glucose-6-phosphate enzyme activity, thereby affecting the glucose biosynthesis pathway. They also demonstrated that AME increases phosphoinositide 3-kinase (PI3K) protein expression, which may improve insulin signal transduction disorders through the PI3K/protein kinase B (Akt) signaling pathway, thereby alleviating insulin resistance (Ke et al., 2013). In type 2 diabetes model (T2DM) rats, AME reduces the release of TNF-α, upregulates glucose transporter 2 expression, enhances the absorption and utilization of blood glucose by the liver and skeletal muscle, and reduces insulin resistance through the PI3K/Akt/mechanistic target of rapamycin (mTOR) and PPARγ signaling pathways (Zhang et al., 2019).

At a dose of 60 mg/kg AME can repair damaged pancreatic tissues in mice with diabetes and enhanced pancreatic islet β cell function (Zheng et al., 2008). The molecular structure of AME can stably bind to the structure of human islet amyloid polypeptide (hIAPP), thereby interfering with the peptide assembly and abnormal folding process, separating hIAPP fibrils into small oligomers and particles, and reducing the cytotoxicity induced by hIAPP oligomerization (Xu et al., 2022). These results indicate that AME can regulate blood lipids and blood glucose from multiple perspectives and different mechanisms and can alleviate various injuries.

#### 3.1.4 Promoting vascular repair

Endothelial growth factor (VEGF) is a key factor in the early formation of blood vessels and a highly selective mitogen for ECs, promoting the proliferation and migration of ECs to promote angiogenesis (Wiszniak and Schwarz, 2021). AME promotes the proliferation of HUVECs in the mitotic S phase in a dose-dependent manner, and upregulates the expression of VEGF, indicating that it plays a role in repairing vascular endothelial cell damage (Zheng et al., 2011). However, under pathological conditions, VEGF increases the instability of atherosclerotic plaques by promoting angiogenesis and inflammatory infiltration, leading to plaque shedding (Qin et al., 2021). Using two different experimental models, it was found that AME can specifically bind to members of the VEGF family, VEGF-A and placental growth factor-1 (PIGF-1), preventing them from further binding to their receptors and inhibiting endothelial cell migration and capillary-like tube production induced by VEGF-A and PIGF-1, thereby inhibiting the growth and formation of vessels (Tarallo et al., 2011). The abnormal proliferation and migration of VSMCs can lead to vascular lesions, which are the hallmark of atherosclerosis, vascular intimal hyperplasia, and arterial stenosis. AME can inhibit the ox-LDL-induced transformation of VSMCs to foam cells through the CD-36/PPARγ signaling pathway, and can inhibit VSMC migration, thereby promoting vascular repair (Zhuang, 2020).

#### 3.1.5 Improving blood circulation

The vasodilator effect of AME may be associated with the muscarinic receptor, the β-adrenergic receptor, and the endothelium-derived vasodilator factor. NO is produced by the catalytic action of iNOS on L-arginine. The vasodilator effect of NO is mediated through the increase of guanosine 3′,5′-cyclic monophosphate (cGMP) levels in smooth muscle. When the vascular endothelium experiences dysfunction, the release of NO decreases, and the vasoconstriction produced by directly activating vascular smooth muscle further reduces the production of NO (Borow et al., 2015). AME has a significant vasodilator effect; however, after injury to the vascular endothelium of injured rats, its vasodilator effect is inhibited, suggesting that AME may act on the vascular endothelium. The vasodilator effect of AME is also inhibited by NO inhibitors in intact vascular endothelium but is not affected by the addition of propranolol hydrochloride and atropine, suggesting that AME may produce a vasodilator effect by affecting the release of NO from the vascular endothelium (Xun and Yin, 2009). iNOS inhibitors can block the vasodilator effect of AME. Guanylate cyclase inhibitors also block the vasodilator effect of AME. Based on a series of experiments, they concluded that AME activates the Ca^2+^-dependent K^+^ channel of ECs, affecting the NO-cGMP signaling pathway, thereby relaxing the vascular smooth muscle and causing vasodilation (Kang et al., 2004).

After the rupture of atherosclerotic plaques, the activation of platelets and thrombin promotes thrombus formation, leading to vascular blockage and interruption of circulation (Ahmed et al., 2020). Thrombin is a serine protease that plays a significant role in the coagulation cascade, thrombus formation, and platelet activation (Brummel et al., 2002). Therefore, drugs that act on thrombin can alleviate disease progression caused by atherosclerosis. AME in (*St. John’s Wort* Diosc.) can inhibit the activity of human thrombin in a dose-dependent manner (Wei et al., 2019). The study compared the effects of 16 major components in *Ginkgo biloba* on thrombin and found that AME has a high affinity for human thrombin and is a strong human thrombin inhibitor. Subsequent molecular docking experiments revealed that it can primarily bind to key amino acids in the active site of thrombin through salt bridges and hydrogen bonds (Chen et al., 2019). Using an acute rat blood stasis model, it was demonstrated that AME can reduce plasma fibrinogen levels to prolong coagulation time, thereby improving blood circulation (Xi et al., 2020). Furthermore, AME has been shown to inhibit platelet aggregation induced by adenosine diphosphate and arachidonic acid, but has no effect on platelet aggregation induced by thrombin (Zhang et al., 2018). The mechanism underlying these effects warrant further study. These results suggest that AME could serve as a natural drug for targeting thrombin and platelets.

### 3.2 Effect of AME on cardiocerebrovascular diseases

#### 3.2.1 Limiting myocardial ischemic injury, myocardial infarction and myocardial fibrosis

Myocardial ischemic injury can lead to myocardial infarction, and myocardial fibrosis may occur after infarction, a process that further affects heart function. If left untreated, myocardial ischemia can lead to damage of the heart, ultimately increasing the risk of developing myocardial infarction over time. Patients who have had a myocardial ischemia often have persistently elevated TNF-α levels. (Ridker et al., 2000). Overexpression of TNF-α, IL-1β, and IL-6 can amplify the harmful effects of inflammation by inducing cell apoptosis. Therefore, inhibiting these inflammatory factors is a promising strategy to prevent the escalation of myocardial ischemic injury. (Kumari et al., 2024).

Ischemic reperfusion injury experiments in mice, have shown that AME significantly reduces the levels of serum myocardial enzymes, specifically lactate dehydrogenase and creatine kinase MS isoenzyme, indicating the protective effects of AME on H9c2 myocardial cells. AME was also shown to significantly reduce the levels of IL-1β, IL-6, and TNF-α in cell supernatants, inhibiting cell apoptosis after myocardial ischemia-reperfusion injury in rats and reducing the size of the myocardial infarction area (Li et al., 2021). It was discovered that AME improved myocardial ischemia-reperfusion injury by modulating the PI3K/Akt-NF-κB signaling pathway, enhancing the phosphorylation of Akt and inhibiting the phosphorylation of NF-κB, reducing cardiac cell apoptosis, and lowering the release of associated inflammatory factors (Li, 2020). In the myocardial infarction model, AME can reduce the values of left ventricular end-systolic diameter and left ventricular end-diastolic diameter, and increase the value of left ventricular ejection fraction, indicating that AME can effectively improve the cardiac function of rats after myocardial infarction. AME can also reduce the expression of carboxy terminal peptide of type I procollagen and the amino terminal peptide of type III procollagen in rats after myocardial infarction, indicating that AME can inhibit myocardial fibrosis after myocardial infarction. The mechanism may involve downregulation of matrix metalloproteinase MMP-2 and transforming growth factor TGF-β1 expression (Chen et al., 2023).

#### 3.2.2 Reducing cardiac insufficiency

AME can improve adriamycin (DOX)-induced cardiac dysfunction and reduce myocardial injury. Furthermore, AME can significantly reduce the expression of cell pyroptosis-related proteins, such as NLRP3, cleaved caspase-1, and cleaved gasdermin D, thereby inhibiting myocardial cell pyroptosis without affecting the expression of apoptosis-related proteins. The primary mechanism of AME action is through the inhibition of the stimulator of interferon genes (STING)/NLRP3 inflammasome signaling pathway (Fang G. et al., 2023). The effect of AME on DOX-induced cardiotoxicity was investigated from several perspectives, including pathological characterization, antioxidant stress, mitochondrial function recovery, anti-inflammatory effects, and apoptosis inhibition. It was found that AME increases heart weight to reverse DOX-induced heart atrophy, ameliorates oxidative stress-induced cardiac injury by reducing MDA, inhibits NADPH oxidase (NOX) expression, increases SOD levels, upregulates the expression of myocardial mitochondrial-related genes nuclear respiratory factor-1 (*NRF-1*) and mitochondrial transcription factor A (*TFAM*) to address mitochondrial dysfunction, decreases IL-6 and NF-κB expression to exert anti-inflammatory effects, upregulates heat shock protein 27 (HSP-27) and downregulates fas ligand (Fasl) expression to influence the process of cell apoptosis (Alherz et al., 2022). Through comparing effects of different dietary supplements on rat atria, it was discovered that AME and quercetin might be the pharmacologically active components of GBE in terms of its positive inotropic and chronotropic effects. Moreover, 10–50 μg/mL of AME can significantly increase the heart rate of rat atria without altering myocardial contractility (Kubota et al., 2002). However, other studies have shown that AME can inhibit the activity of cAMP diesterase, thereby enhancing myocardial contractility and dilating peripheral vessels, further promoting blood flow in the body and reducing pressure on the heart and vessels (Saponara and Bosisio, 1998). This suggests that AME is a potential drug for the treatment of heart failure.

#### 3.2.3 Improving cardiovascular function

It was discovered that AME exerts a protective effect on cardiovascular dysfunction through several mechanisms: (1) Ultrasonic electrocardiographic evaluations have shown that AME can inhibit the increase in left ventricular internal diameter and the thickness of the posterior wall during diastole, reduce left ventricular mass, alter the ejection fraction and relative wall thickness, and inhibit the increase in cardiac stiffness and left ventricular wet weight induced by an HFFD. (2) In settings of oxidative stress, AME can modify the levels of oxidative stress markers such as thiobarbituric acid reactive substances, glutathione (GSH), SOD, catalase (CAT) in plasma, and can inhibit the increase in NOX in the heart, thereby reducing the degree of oxidative stress-induced cardiac injury. (3) In rats fed a HFFD, AME inhibits the increased expression of angiotensin (Ang) II receptors, angiotensin-1A receptor and the decreased expression of angiotensin type 2 receptors in the renin-angiotensin system and can significantly inhibit the increase in blood pressure. (4) AME inhibits phenylephrine-induced aortic vasoconstriction and increased acetylcholine-induced vascular relaxation. The mechanism of action of AME may involve the regulation of NOX, thereby modulating angiotensin II cell signaling and oxidative stress (Qin et al., 2018).

#### 3.2.4 Limiting ischemic brain damage

Ischemic stroke is one of the most common brain diseases, accounting for 85% of cerebrovascular diseases (Oliveira et al., 2023). Research has shown that AME can protect the brain from hypoxic-ischemic injury from multiple perspectives. First, administration of AME to rats with hypoxic-ischemic brain injury reduced brain tissue damage in the forebrain by 50%. Second, *in vitro* experiments showed that AME exhibits excellent neuroprotective effects against DNA damage, mitochondrial damage, and NO-induced injury. *In vivo* experiments demonstrated that AME inhibits caspase 3-induced cell pyroptosis in a dose-dependent manner, decreases the expression of iNOS and COX-2, and suppresses the production of inflammatory factors such as IL-1β and TNF-α stimulated by LPS, thereby reducing the damage caused to microglia by these inflammatory factors (Shin et al., 2006). In a study, a model of left common carotid artery occlusion stroke to investigate the protective effects of AME on cerebral ischemia/reperfusion injury in rats. The findings indicated that after ischemia/reperfusion injury, AME significantly reduced the neurological deficit scores, improved motor coordination ability and spontaneous activity, reduced the levels of TNF-α, IL-1β, and IL-6, inhibited the NF-κB signaling pathway, reduced caspase-3 to block cell apoptosis, increased the levels of TNF receptor-associated factor family member-associated NF-κB activator-binding kinase 1 and interferon β, reduced MDA levels, and increased GSH and CAT levels in the brain. The mechanism of AME may be mediated by high mobility group box protein B1 (HMGB1) through the toll-like receptor-4 (TLR4)/NF-κB signaling pathway (Saeedan et al., 2023). These experimental phenomena and mechanisms indicate that AME has a good protective effect on ischemic brain injury.

### 3.3 Effect of AME on neurological diseases

#### 3.3.1 Preventing Alzheimer’s disease

Alzheimer’s disease (AD) is a neurodegenerative disorder characterized by the accumulation of β-amyloid (Aβ) peptides, neurofibrillary tangles formed by hyperphosphorylated tau proteins, abnormal oxidative stress damage, inflammatory responses, and neurotransmitter disorders in affected brain regions (Breijyeh and Karaman, 2020). It is commonly treated with acetylcholinesterase (AChE) inhibitors and N-methyl-D-aspartate receptor antagonists. Although these medications can alleviate clinical manifestations, they do not reverse cognitive impairment, and are often accompanied by common side effects, including gastrointestinal symptoms, confusion, dizziness, and headaches. (Chin et al., 2022).

Overproduction of Aβ leads to the formation of neurofibrillary plaques in the brain, which subsequently accumulate in the blood vessels to cause cerebral amyloid angiopathy (Han et al., 2022). Therefore, inhibiting the production of Aβ and promoting its clearance are crucial for improving AD. In a study, researchers used the fluorescent dye thiazine 1,3,5-tetracarboxylic acid to investigate the inhibitory effect of various flavonoid compounds on Aβ aggregation and the structure-activity relationship of promoting Aβ fibril disaggregation. The results indicated that AME could inhibit the formation of Aβ_1-42_ fibrils, had a better affinity for Aβ_1-42_ fibrils than for Aβ_1-40_ fibrils. The hydrophilic group of AME was the key group for its function. Furthermore, among flavonoid compounds with different structures, AME with four hydroxyl groups could most effectively inhibit Aβ fibrillation and promote the disaggregation of pre-formed Aβ fibrils (Choi et al., 2020). Another study showed that increasing the number of methoxy groups on the AME parent structure weakened the inhibitory activity of AME on Aβ_40_, and the connection of single bonds also affected the inhibitory activity (Sirimangkalakitti et al., 2019). A study elucidated how AME inhibits the aggregation of Aβ_42_ peptide at the atomic level. AME bound to aromatic residues in the N-terminal of Aβ_42_ peptide, forming a stable π–π interaction, which destabilized the fibril structure. Subsequently, AME utilized its hydrogen bond donor/acceptor specificity to disrupt the hydrogen bond potential of the fibril peptide backbone, thereby disrupting the fibril structure and promoting Aβ fibril disaggregation (Windsor et al., 2021).

A study explained the anti-AD function of AME from the perspective of receptor-mediated endocytosis for Aβ clearance. AME can significantly increase Aβ uptake by mouse N2a neural cells through class A scavenger receptors, and then enter the lysosome within the cell, where it is degraded by relevant enzymes without causing cytotoxicity. The hydroxyl group in the molecular structure of AME enhances Aβ uptake, whereas the substitution of methyl groups decreases its uptake effect (Han et al., 2022). Another study identified AME from a library of polyphenolic compounds that can increase the activity and expression of neuropeptidase, an Aβ degradation enzyme, thereby delaying the progression of AD. Among them, the double-bond chemical structure in the C ring of the AME structure can significantly enhance neuropeptidase activity (Hori et al., 2023).

Research indicated that among various flavonoid compounds, AME exhibited the strongest neuroprotective effect, significantly inhibiting ROS-induced oxidative stress damage in SHSY5Y neuroblastoma cells. AME reduced DNA damage induced by etoposide DNA damage, whereas other flavonoid compounds increased cell death caused by DNA damage. Moreover, at a concentration of 2 μM, AME was most effective at reducing cytotoxicity induced by the Aβ_25-35_ peptide in rat PC12 cells, indicating the therapeutic potential of AME in neurodegenerative diseases (Kang et al., 2005). Futher studies found that AME could differentially protect SH-SY5Y neural cells from various cytotoxic factors such as hydrogen peroxide, okadiac acid, and Aβ_25-35_. Specifically, AME was able to inhibit okadiac acid-induced tau protein hyperphosphorylation, restore mitochondrial membrane potential to inhibit cell apoptosis, and protect microtubule structure and mitochondrial function. Furthermore, AME exhibited strong antioxidant capabilities against mitochondrial dysfunction and ROS-induced oxidative stress damage. AME also bound to β-secretase to inhibit its activity, thereby preventing the degradation of Aβ protein precursor to produce large amounts of Aβ (Zhang, 2014). In models of AD, AME could activate nuclear factor erythroid 2-related factor-2 (Nrf2) through affecting the adenosine monophosphate-activated protein kinase (AMPK)/GSK-3-β signaling pathway to exert antioxidant stress effects, ameliorating Aβ-induced neural function deficits and neuronal apoptosis (Chen et al., 2018). Although studies have shown that GSK-3-β is involved in the regulation of Nrf2 by AME, further verification is required to determine the mechanism by which AME affects GSK-3-β signaling-mediated tau phosphorylation. Tt was demonstrated through multiple experiments that AME, as a bifunctional chelator, can bind to various Aβ aggregates with high affinity and reduce their induced cytotoxicity. Additionally, AME can effectively chelate with Cu^2+^ and exhibit strong antioxidant activity, thereby preventing the formation of soluble Aβ oligomers and ROS-induced by metal ions in the brain (Sun et al., 2020).

Autophagy can degrade and recycle proteins produced by misfolding and damaged organelles, thereby maintaining protein homeostasis. The activation of autophagy in cells can effectively eliminate the accumulation of Aβ, thereby reducing the progression of AD (Zhang Z. et al., 2021). AME can improve the memory and cognitive impairment induced by Aβ_25-35_ in the brains of mice and alleviate inflammation, oxidative stress, and the immune response in the hippocampus. Additionally, it can enhance autophagy by binding to multiple amino acid residues of the mTOR protein, thereby inhibiting further phosphorylation of mTOR and exerting a neuroprotective effect against AD (Cao et al., 2021). Experiments were conducted on neuronal cell damage induced by Aβ_1-42_. It was found that AME can improve neuronal dysfunction induced by Aβ_1-42_ in rats and inhibit NLRP3 inflammasome-induced pyroptosis by regulating AMPK/GSK-3-β signaling, thereby ameliorating the neurotoxicity caused by Aβ_1-42_ in AD (Zhao et al., 2019).

Using the scopolamine-induced dementia mouse model, it was discovered that oral administration of AME can inhibit the activity of AChE and increase acetylcholine levels. Furthermore, it can improve the oxidative stress damage induced by scopolamine in mice, mainly by reducing MDA levels and increasing GSH activity, thereby ameliorating cognitive impairment (Ishola et al., 2013a). Addtionally, a study collected 50 flavonoid compounds with therapeutic potential for AD. Molecular docking experiments were conducted with the human α7 nicotinic acetylcholine receptor (α7nACHR) and found that after binding with AME, it exhibited the best affinity and good stability parameters (Singh et al., 2023). However, the effectiveness of AME as an α7nACHR agonist for treating AD requires further verification.

#### 3.3.2 Treating Parkinson’s disease

In Parkinson’s disease (PD), melanin is released as neurons degenerate. Subsequently, it is recognized and ingested by microglia, leading to inflammatory damage. In patients with PD, the breakdown of dopaminergic neurons can be attributed to chronic neuroinflammation, which leads to the production and accumulation of Lewy bodies in the compact region of the substantia nigra in the brain. The main pathological features of PD are damage to dopaminergic neurons in the midbrain substantia nigra and the establishment of diffuse Lewy bodies, which subsequently lead to motor neuron dysfunction. Therefore, taking measures to inhibit neuroinflammation may be beneficial for alleviating PD clinical manifestations (Sharma et al., 2023).

Microglia, analogous to macrophages within the brain, can be activated by various stimuli to release a range of inflammatory mediators, thereby initiating an inflammatory response that may result in neural damage (Cai et al., 2022). AME has been shown to improve motor dysfunction and anxiety-like and depressive-like behaviors in the LPS-induced PD rat model. Mechanistic studies revealed that AME effectively inhibits the expression of pro-inflammatory factors (TNF-α, IL-1β, IL-6, iNOS, COX-2) and enhances the expression of anti-inflammatory factors (IL-4, TGF-β, Arg-1, CD206), indicating that AME promotes the polarization of microglia towards the M2 anti-inflammatory phenotype. Furthermore, AME significantly ameliorates the reduction in tyrosine hydroxylase-positive neurons and the increase in α-synuclein expression observed in the model, suggesting that AME exerts neuroprotective effects in the LPS-induced PD model (Liu et al., 2024). It has been discovered that after LPS stimulation2 of rat astrocytoma cells (C6), AME significantly inhibited the production of nitrites, ROS, MDA, and TNF-α, upregulated GSH levels, and reduced intracellular oxidative stress (Ishola et al., 2013b). Activated ERK1/2 promotes cell death due to oxidative toxicity, but AME can alleviate oxidative stress damage caused by glutamate and ROS in HT22 neuronal cells by maintaining the activity of antioxidant enzymes and inhibiting ERK1/2 activity (Jeong et al., 2014). Molecular docking studies have revealed that AME exhibits strong binding affinity with the glutathione peroxidase 4 (GPX4) protein, which is involved in ferroptosis (Xiong et al., 2024). It was reported that AME can inhibit inflammation induced by ferroptosis in HT22 cells triggered by homocysteine. However, it is important to note that AME treatment results in decreased expression levels of both solute carrier family 7 member 11 (SLC7A11) and GPX4. Given that the SLC7A11/GPX4 signaling pathway requires the upregulation of these proteins to effectively suppress ferroptosis, the observed reduction in their expression following AME treatment may affect its efficacy in preventing ferroptosis (Wang et al., 2024).

*In vivo* and *in vitro* PD model studies demonstrated that AME can significantly reduce the expression of glial fibrillary acidic protein and lba1 markers in glial cells under inflammatory conditions, decrease the activation of caspase-3 and p21, and reduce the expression levels of IL-1β and iNOS and increase the Bcl-2/Bax ratio, through the PI3K/Akt and ERK signaling pathways. In this study, in a vitro mouse model induced by methyl-4-phenyl-1,2,3,6-tetrahydropyridine (MPTP), AME was able to protect dopaminergic neurons and reduce striatal fiber loss (Cao et al., 2017). These findings suggest that AME can exert beneficial effects on dopaminergic neurons and glial cells, thereby ameliorating the clinical manifestations of PD. Furthermore, studies have shown a deregulated angiotensin-converting enzyme (ACE)/Ang II/angiotensin II receptor-1 (AT1R) axis is activated at the onset of PD, leading to free radical damage, cell apoptosis, and neuronal disruption. AME can bind to the mitochondrial assembly receptor (MASR) protein and activate the ACE2/Ang (1–7)/MASR, thereby neutralizing the neurodegenerative changes triggered by the ACE/Ang II/AT1R axis (Bhadauriya et al., 2023).

#### 3.3.3 Treating epilepsy

The development of inflammation is closely related to the onset of epilepsy and its clinical progression. Brain inflammation can promote neuronal excitability, reducing the seizure threshold, thereby triggering seizures. Therefore, anti-inflammatory therapy can be used to prevent and treat seizures (Alsaegh et al., 2021). Studies found that AME improve pentetrazole-induced cognitive dysfunction by inhibiting the NLRP3 inflammasome, reducing the susceptibility to seizures and apoptosis in hippocampal neurons of mice. In this study, AME can also inhibit the mRNA expression of NLRP3, ASC, and caspase-1 in BV2 microglia induced by LPS, reducing the expression of inflammatory factors IL-1β, IL-18, and TNF-α. However, in the absence of inflammation, administration of AME to mice did not affect the expression of these proteins and inflammatory factors (Rong et al., 2019). Research indicated that AME can inhibit the activation and nuclear translocation of NF-κB p65 subunit in mice, thereby inhibiting the NF-κB signaling pathway to reduce the injury of hippocampal CA1 neural cells. AME can also reduce the production of inflammatory mediators NO and PGE-2, and inflammatory factors IL-6 and IL-β in neural cells. Furthermore, histological analysis found that AME can protect neurons after status epilepticus, inhibit the excessive discharge of hippocampal neurons, and shorten the duration of seizures (Zhang et al., 2015). These results indicate that AME has a good protective effect on hippocampal neuronal injury caused by epilepsy and has good anti-inflammatory effects. A study employed various computational methods and found that AME exhibits superior affinity for α-amino-3-hydroxy-5-methyl-4-isoxazolepropionic acid (AMPA) and voltage-gated sodium ion channels (VGSC) receptors compared to phenytoin. Additionally, physicochemical and pharmacokinetic studies indicate that AME is suitable for oral administration, demonstrating favorable intestinal permeability and the ability to cross the blood-brain barrier, with no significant risk of toxicity (Salaria et al., 2024). These findings suggest that AME is a promising candidate for further research as a potential treatment for epilepsy.

## 4 Conclusion and future perspective

AME has been reported in multiple studies as the primary active pharmacological component from its plant sources, with a wide range of pharmacological effects (Yu et al., 2017; Li et al., 2021; Zhang et al., 2015). Due to the unique chemical structure of AME, its biological activity can be significantly influenced by multiple factors. Further studies could elucidate how various factors, such as photooxidation, affect the structural stability of AME. Additionally, pharmacokinetic studies have demonstrated that AME undergoes rapid metabolism in the body, resulting in low bioavailability. Therefore, modifying the natural structure or developing suitable drug formulations of AME is crucial for improving its therapeutic properties and enhancing its bioavailability.

The anti-inflammatory, antioxidant, and lipid-lowering effects of AME are notable, and recent studies have demonstrated its application in various cardiac and neurological diseases, suggesting that it may have significant anti-atherosclerotic effects. However, most of the current data on AME are derived from *in vitro* cell experiments, with limited *in vivo* animal testing. More comprehensive animal experiments are required to validate its pharmacological activity. One study has found that AME may have hepatotoxic and nephrotoxic effects (Li D et al., 2019). This warrants further investigation.

AME exhibits significant therapeutic effects on various cardiocerebrovascular diseases and neurological disorders, potentially functioning through multiple mechanisms. All these disease processes are accompanied by inflammatory responses, which are triggered by multiple factors. AME can affect multiple inflammatory mediators and mechanisms, suggesting that it has considerable potential for treating these diseases. However, the specific mechanisms of action involved in the effects of AME on inflammatory diseases require further research. Hemodynamic factors such as hypertension cannot be ignored in the development of cardiocerebrovascular; however, the mechanisms and targets by which AME counters the effects of these factors remain unclear. Currently, there remains controversy regarding whether AME can exert a therapeutic effect on AD. Furthermore, the specific mechanisms by which AME influences the cellular uptake and clearance of Aβ also require further validation research.

In summary, AME exhibits multiple activities, indicating its potential as a natural drug for treating cardiocerebrovascular diseases and neurological disorders. Further studies on its pharmacokinetics and toxicology are required to ensure its safety and efficacy.

## Glossary

AME: Amentoflavone
AGEs: Advanced glycation end-products
ASK1: Apoptosis signal regulating kinase 1
Akt: Protein kinase B
AD: Alzheimer’s disease
Aβ: β-amyloid
AChE: Acetylcholinesterase
Ang: Angiotensin
ACE: Angiotensin-converting enzyme
AMPK: Adenosine monophosphate-activated protein kinase
AT1R: Angiotensin II receptor-1
*Arg1*: Arginase 1
AUC(0-t): Area under the concentration-time cure
AUC(0-∞): From time zero to all original drug elimination
AMPA: α-amino-3-hydroxy-5-methyl-4-isoxazolepropionic acid
α7nACHR: α7 nicotinic acetylcholine receptor
***C***_**max**_: Maximum blood concentration
*CL/F*: Clearance
COX-2: Cyclooxygenase-2
CD36: Cluster of differentiation 36
C/EBP: Transcription factor CCAAT enhancer binding protein
CAT: Catalase
cGMP: Guanosine 3’,5’-cyclic monophosphate
caspase: Cysteinyl aspartate specific protease
DPPH: 1,1-diphenyl-2 trinitrophenylhydrazine
DOX: Adriamycin
ECs: Endothelial cells
ET-1: Endothelin-1
ERK: Extracellular signal-regulated kinase
Fasl: Fas ligand
*F*: Bioavailability
*Fizz1*: Found in inflammatory zone
GSK-3-β: Glucose synthesis kinase-3-β
GBE: *Ginkgo biloba* extract
GSH: Glutathione
GPX4: Glutathione peroxidase 4
HMGB1: High mobility group box protein B1
HSP-27: Heat shock protein 27
HFFD: High fructose and fat diet
HPLC: High-performance liquid chromatography
HUVECs: Human umbilical vein endothelial cells
hIAPP: Human islet amyloid polypeptide
IL: Interleukin
IκB: Inhibitor of NF-κB
iNOS: Nitric oxide synthase
LPS: Lipopolysaccharide
LDL: Low-density lipoprotein
**MRT**_**0-∞**_: Mean residence time
**MRT**_**0-t**_: Average retention time for a certain period of time
MMPs: Matrix metalloproteinases
MCE: Mitotic clonal expansion phase
MDA: Malondialdehyde
MAPK: Mitogen-activated protein kinase
mTOR: Mechanistic target of rapamycin
MPTP: Methyl-4-phenyl-1,2,3,6-tetrahydropyridine
MASR: Mitochondrial assembly receptor
MIF: Macrophage migration inhibitory factor
NLRP3: NOD-like receptor thermal protein domain associated protein 3
NOX: NADPH oxidase
NF-κB: Nuclear factor-kappa B
NO: Nitric oxide
Nrf2: Nuclear factor erythroid 2-related factor-2
NRF-1: Nuclear respiratory factor-1
ox-LDL: Oxidized low-density lipoprotein
PGE-2: Prostaglandin E-2
PPAR: Peroxisome proliferator-activated receptor
PI3K: Phosphoinositide 3-kinase
PD: Parkinson’s disease
PIGF-1: Placental growth factor-1
ROS: Reactive oxygen species
SOD: Superoxide dismutase
STING: Stimulator of interferon genes
SLC7A11: Solute carrier family 7 member 11
TG: Triglycerides
Trx: Thioredoxin
TrxR-1: Thioredoxin reductase-1
***T***_**max**_: Time to peak concentration
***T***_***1/2***_: Biological half-life
T2DM: Type 2 diabetes model
TNF: Tumor necrosis factor
TGF: Transforming growth factor
TLR4: Toll-like receptor-4
TFAM: Mitochondrial transcription factor
UGT1A: Uridine diphosphate glucuronosyltransferase 1 family, polypeptide A
V/F: Apparent distribution volume
VSMCs: Vascular smooth muscle cells
VEGF: Endothelial growth factor
VGSC: Voltage-gated sodium ion channels
*Ym1*: Chitinase-like protein 3

## References

[B1] Ahmed M. U. Kaneva V. Loyau S. Nechipurenko D. Receveur N. Le Bris M. (2020). Pharmacological blockade of glycoprotein VI promotes thrombus disaggregation in the absence of thrombin. Arterioscler. Thromb. Vasc. Biol. 40 (9), 2127–2142. 10.1161/ATVBAHA.120.314301 32698684

[B2] Alherz F. A. El-Masry T. A. Negm W. A. El-Kadem A. H. (2022). Potential cardioprotective effects of Amentoflavone in doxorubicin-induced cardiotoxicity in mice. Biomed. Pharmacother. 154, 113643. 10.1016/j.biopha.2022.113643 36942597

[B3] Alkadi K. A. A. Ashraf K. Adam A. Shah S. A. A. Taha M. Hasan M. H. (2021). *In vitro* cytotoxicity and anti-inflammatory cytokinine activity study of three isolated novel compounds of *Prismatomeris glabra* . J. Pharm. Bioallied Sci. 13 (1), 116–122. 10.4103/jpbs.JPBS_279_19 34084057 PMC8142914

[B4] Alsaegh H. Z. Eweis H. S. A. E-K. Kamel F. O. (2021). Pathophysiological cascade of events leading to epilepsy: role of inflammation. J. Pharm. Res. Int. 32 (48), 74–84. 10.9734/jpri/2020/v32i4831127

[B5] Bajpai V. K. Park I. Lee J. Shukla S. Nile S. H. Chun H. S. (2019). Antioxidant and antimicrobial efficacy of a biflavonoid, amentoflavone from *Nandina domestica in vitro* and in minced chicken meat and apple juice food models. Food Chem. 271, 239–247. 10.1016/j.foodchem.2018.07.159 30236673

[B6] Banerjee T. Valacchi G. Ziboh V. A. van der Vliet A. (2002). Inhibition of TNFalpha-induced cyclooxygenase-2 expression by amentoflavone through suppression of NF-kappaB activation in A549 cells. Mol. Cell Biochem. 238 (1-2), 105–110. 10.1023/a:1019963222510 12349896

[B7] Bhadauriya P. Varshney V. Goyal A. (2023). Molecular docking-based identification of potential natural neuroprotective molecules for Parkinson's disease. Chem. Biodivers. 20 (10), e202300979. 10.1002/cbdv.202300979 37608470

[B8] Bhattacharya P. Mandal A. (2024). Identification of amentoflavone as a potent SARS-CoV-2 M^pro^ inhibitor: a combination of computational studies and *in vitro* biological evaluation. J. Biomol. Struct. Dyn. 1, 1–19. 10.1080/07391102.2024.2304676 38263736

[B9] Borow K. M. Nelson J. R. Mason R. P. (2015). Biologic plausibility, cellular effects, and molecular mechanisms of eicosapentaenoic acid (EPA) in atherosclerosis. Atherosclerosis 242 (1), 357–366. 10.1016/j.atherosclerosis.2015.07.035 26253795

[B10] Breijyeh Z. Karaman R. (2020). Comprehensive review on Alzheimer's disease: causes and treatment. Molecules 25 (24), 5789. 10.3390/molecules25245789 33302541 PMC7764106

[B11] Brummel K. E. Paradis S. G. Butenas S. Mann K. G. (2002). Thrombin functions during tissue factor-induced blood coagulation. Blood 100 (1), 148–152. 10.1182/blood.v100.1.148 12070020

[B12] Cai Y. Liu J. Wang B. Sun M. Yang H. (2022). Microglia in the neuroinflammatory pathogenesis of Alzheimer's disease and related therapeutic targets. Front. Immunol. 13, 856376. 10.3389/fimmu.2022.856376 35558075 PMC9086828

[B13] Cao B. Zeng M. Zhang Q. Zhang B. Cao Y. Wu Y. (2021). Amentoflavone ameliorates memory deficits and abnormal autophagy in aβ_25-35_-induced mice by mTOR signaling. Neurochem. Res. 46 (4), 921–934. 10.1007/s11064-020-03223-8 33492604

[B14] Cao Q. Qin L. Huang F. Wang X. Yang L. Shi H. (2017). Amentoflavone protects dopaminergic neurons in MPTP-induced Parkinson's disease model mice through PI3K/Akt and ERK signaling pathways. Toxicol. Appl. Pharmacol. 319, 80–90. 10.1016/j.taap.2017.01.019 28185818

[B15] Carracedo M. Artiach G. Arnardottir H. Bäck M. (2019). The resolution of inflammation through omega-3 fatty acids in atherosclerosis, intimal hyperplasia, and vascular calcification. Semin. Immunopathol. 41 (6), 757–766. 10.1007/s00281-019-00767-y 31696250 PMC6881483

[B16] Chen B. Xu D. Li Z. Jing Y. Lin L. Li S. (2022). Tissue distribution, excretion, and interaction with human serum albumin of total bioflavonoid extract from *Selaginella doederleinii* . Front. Pharmacol. 13, 849110. 10.3389/fphar.2022.849110 35571075 PMC9099209

[B17] Chen C. L i B. Cheng G. Yang X. Zhao N. Shi R. (2018). Amentoflavone ameliorates aβ_1-42_-induced memory deficits and oxidative stress in cellular and rat model. Neurochem. Res. 43 (4), 857–868. 10.1007/s11064-018-2489-8 29411261

[B18] Chen G. Han Y. He W. Liang F. (2016). Amentoflavone protects against high fat-induced metabolic dysfunction: possible role of the regulation of adipogenic differentiation. Int. J. Mol. Med. 38 (6), 1759–1767. 10.3892/ijmm.2016.2772 27748827 PMC5117752

[B19] Chen T. R. Wei L. H. Guan X. Q. Huang C. Liu Z. Y. Wang F. J. (2019). Biflavones from *Ginkgo biloba* as inhibitors of human thrombin. Bioorg Chem. 92, 103199. 10.1016/j.bioorg.2019.103199 31446241

[B20] Chen W. M. Chen J. M. Jian M. H. (2023). Effect of amentoflavone on myocardial fibrosis in rats with acute myocardial infarction. J. Zunyi Med. Univ. 46 (07), 646–651+656. 10.14169/j.cnki.zunyixuebao.2023.0103

[B21] Chin E. Jaqua E. Safaeipour M. Ladue T. (2022). Conventional versus new treatment: comparing the effects of acetylcholinesterase inhibitors and N-Methyl-D-Aspartate receptor antagonist with aducanumab. Cureus 14 (11), e31065. 10.7759/cureus.31065 36475205 PMC9719396

[B22] Cho S. Lee H. Han J. Lee H. Kattia R. O. Nelson Z. V. (2021). *Viburnum stellato-Tomentosum* extract suppresses obesity and hyperglycemia through regulation of lipid metabolism in high-fat diet-fed mice. Molecules 26 (4), 1052. 10.3390/molecules26041052 33671428 PMC7922011

[B23] Choi E. Y. Kang S. S. Lee S. K. Han B. H. (2020). Polyphenolic biflavonoids inhibit amyloid-beta fibrillation and disaggregate preformed amyloid-beta fibrils. Biomol. Ther. Seoul. 28 (2), 145–151. 10.4062/biomolther.2019.113 31697876 PMC7059817

[B24] Duan S. Jia J. F. Hong B. Zhou J. Zhang Y. Ge F. (2022). Assessment of amentoflavone loaded sub-micron particle preparation using supercritical antisolvent for its antitumor activity. Curr. Drug Deliv. 19 (1), 41–48. 10.2174/1567201818666210810142750 35135460

[B25] Dutta S. Singhal A. K. Suryan V. Chandra N. C. (2023). Obesity: an impact with cardiovascular and cerebrovascular diseases. Ind. J. Clin. Biochem. 39, 168–178. 10.1007/s12291-023-01157-w PMC10987439 38577137

[B26] Fang G. Li X. Yang F. Huang T. Qiu C. Peng K. (2023b). Amentoflavone mitigates doxorubicin-induced cardiotoxicity by suppressing cardiomyocyte pyroptosis and inflammation through inhibition of the STING/NLRP3 signalling pathway. Phytomedicine 117, 154922. 10.1016/j.phymed.2023.154922 37321078

[B27] Fang H. Y. Zhao X. N. Zhang M. Ma Y. Y. Huang J. L. Zhou P. (2023a). Beneficial effects of flavonoids on cardiovascular diseases by influencing NLRP3 inflammasome. Inflammopharmacology 31 (4), 1715–1729. 10.1007/s10787-023-01249-2 37261627

[B28] Fang H. Y. Zhuang R. X. Wang F. G. Xi J. J. Zang H. Q. Miao L. B. (2011). Separation and purification of total flavonoids from *Selaginella moellendorfii* hieron by macroporous resin and detected by HPLC. Chin. Arch. Tradit. Chin. Med. 29 (12), 2796–2798. 10.13193/j.archtcm.2011.12.198.fanghy.052

[B29] Feng X. (2020). Study on metabolism of amentoflavone and isoginkgetin *in vitro* and *in vivo* preparation and evaluation of their nanomicelles. Shijiazhuang (Hebei): Hebei Medical University. dissertation.

[B30] Feng X. Chen Y. Li L. Zhang Y. Zhang L. Zhang Z. (2020). Preparation, evaluation and metabolites study in rats of novel amentoflavone-loaded TPGS/soluplus mixed nanomicelles. Drug Deliv. 27 (1), 137–150. 10.1080/10717544.2019.1709920 31913733 PMC6968485

[B31] Feng Y. Wang J. Zhang S. Li Y. Wang B. Zhang J. (2023). Preparation of amentoflavone-loaded DSPE-PEG (2000) micelles with improved bioavailability and *in vitro* antitumor efficacy. Biomed. Chromatogr. 37 (9), e5690. 10.1002/bmc.5690 37337343

[B32] Ferchichi L. Derbré S. Mahmood K. Touré K. Guilet D. Litaudon M. (2012). Bioguided fractionation and isolation of natural inhibitors of advanced glycation end-products (AGEs) from *Calophyllum flavoramulum* . Phytochemistry 78, 98–106. 10.1016/j.phytochem.2012.02.006 22445651

[B33] Ference B. A. Ginsberg H. N. Graham I. Ray K. K. Packard C. J. Bruckert E. (2017). Low-density lipoproteins cause atherosclerotic cardiovascular disease. 1. Evidence from genetic, epidemiologic, and clinical studies. A consensus statement from the European Atherosclerosis Society Consensus Panel. Eur. Heart J. 38 (32), 2459–2472. 10.1093/eurheartj/ehx144 28444290 PMC5837225

[B34] Gan L. Ma J. You G. Mai J. Wang Z. Yang R. (2020). Glucuronidation and its effect on the bioactivity of amentoflavone, a biflavonoid from Ginkgo biloba leaves. J. Pharm. Pharmacol. 72 (12), 1840–1853. 10.1111/jphp.13247 32144952

[B35] Global Burden of Disease Study 2013 Collaborators (2015). Global, regional, and national incidence, prevalence, and years lived with disability for 301 acute and chronic diseases and injuries in 188 countries, 1990-2013: a systematic analysis for the Global Burden of Disease Study 2013. Lancet 386 (9995), 743–800. 10.1016/S0140-6736(15)60692-4 26063472 PMC4561509

[B36] Gui Y. Zheng H. Cao R. Y. (2022). Foam cells in atherosclerosis: novel insights into its origins, consequences, and molecular mechanisms. Front. Cardiovasc Med. 9, 845942. 10.3389/fcvm.2022.845942 35498045 PMC9043520

[B37] Guo Q. Jin Y. Chen X. Ye X. Shen X. Lin M. (2024). NF-κB in biology and targeted therapy: new insights and translational implications. Signal Transduct. Target Ther. 9 (1), 53. 10.1038/s41392-024-01757-9 38433280 PMC10910037

[B38] Han B. H. Cofell B. Everhart E. Humpal C. Kang S. S. Lee S. K. (2022). Amentoflavone promotes cellular uptake and degradation of amyloid-beta in neuronal cells. Int. J. Mol. Sci. 23 (11), 5885. 10.3390/ijms23115885 35682567 PMC9180170

[B39] He C. (2013). The mechanism of Mfn2 Gene to promote cholesterol effulux in vascular smooth muscle cell-derived foam cells. Wuhan (Hubei): Huazhong University of Science and Technology. dissertation.

[B40] Hori Y. Watanabe K. Yassen A. S. A. Shirotani K. Tanaka T. Iwata N. (2023). Enhancement of neprilysin activity by natural polyphenolic compounds and their derivatives in cultured neuroglioma cells. Biol. Pharm. Bull. 46 (3), 446–454. 10.1248/bpb.b22-00833 36858574

[B41] Huang Q. Z. Sun J. (2023). Correlation analysis of blood glucose and lipid levels with lower extremity vascular disease in patients with type 2 diabetes mellitus. Mod. Med. Health Res. Electron J. 7 (18), 16–18. 10.3969/j.issn.2096-3718.2023.18.006

[B42] Huvaere K. Sinnaeve B. Van Bocxlaer J. Skibsted L. H. (2012). Flavonoid deactivation of excited state flavins: reaction monitoring by mass spectrometry. J. Agric. Food Chem. 60 (36), 9261–9272. 10.1021/jf301823h 22889117

[B43] Iadecola C. (2023). The pathobiology of vascular dementia. Neuron 80 (4), 844–866. 10.1016/j.neuron.2013.10.008 PMC3842016 24267647

[B44] Ishola I. O. Chatterjee M. Tota S. Tadigopulla N. Adeyemi O. O. Palit G. (2012). Antidepressant and anxiolytic effects of amentoflavone isolated from *Cnestis ferruginea* in mice. Pharmacol. Biochem. Behav. 103 (2), 322–331. 10.1016/j.pbb.2012.08.017 22944105

[B45] Ishola I. O. Chaturvedi J. P. Rai S. Rajasekar N. Adeyemi O. O. Shukla R. (2013b). Evaluation of amentoflavone isolated from *Cnestis ferruginea* Vahl ex DC (Connaraceae) on production of inflammatory mediators in LPS stimulated rat astrocytoma cell line (C6) and THP-1 cells. J. Ethnopharmacol. 146 (2), 440–448. 10.1016/j.jep.2012.12.015 23376104

[B46] Ishola I. O. Tota S. Adeyemi O. O. Agbaje E. O. Narender T. Shukla R. (2013a). Protective effect of *Cnestis ferruginea* and its active constituent on scopolamine-induced memory impairment in mice: a behavioral and biochemical study. Pharm. Biol. 51 (7), 825–835. 10.3109/13880209.2013.767360 23627469

[B47] Jeong E. J. Hwang L. Lee M. Lee K. Y. Ahn M. J. Sung S. H. (2014). Neuroprotective biflavonoids of *Chamaecyparis obtusa* leaves against glutamate-induced oxidative stress in HT22 hippocampal cells. Food Chem. Toxicol. 64, 397–402. 10.1016/j.fct.2013.12.003 24315869

[B48] Jiang Y. Wang S. Yu M. Wu D. Lei J. Li W. (2020). Ultrasonic-assisted ionic liquid extraction of two biflavonoids from *Selaginella tamariscina* . ACS Omega 5 (51), 33113–33124. 10.1021/acsomega.0c04723 33403273 PMC7774283

[B49] Kang D. G. Yin M. H. Oh H. Lee D. H. Lee H. S. (2004). Vasorelaxation by amentoflavone isolated from *Selaginella tamariscina* . Planta Med. 70 (8), 718–722. 10.1055/s-2004-827201 15326548

[B50] Kang S. S. Lee J. Y. Choi Y. K. Song S. S. Kim J. S. Jeon S. J. (2005). Neuroprotective effects of naturally occurring biflavonoids. Bioorg Med. Chem. Lett. 15 (15), 3588–3591. 10.1016/j.bmcl.2005.05.078 15978805

[B51] Ke Y. Y. Yuan P. P. Zhou Y. Y. Zhang X. Li M. Wang S. (2013). “The intervention experimental study of total flavonoids in *Selaginella tamariscina (Beauv.) Spring* and Amentotaxus Biflavone in HepG2 cells of insulin resistance,” in 2013 China Pharmaceutical Conference and the 13th Chinese pharmacist weekly treatise, 1382–1391.

[B52] Khafagy E. S. Soliman G. A. Shahba A. A. Aldawsari M. F. Alharthy K. M. Abdel-Kader M. S. (2023). Brain targeting by intranasal drug delivery: effect of different formulations of the biflavone “cupressuflavone” from juniperus sabina L. On the motor activity of rats. Molecules 28 (3), 1354. 10.3390/molecules28031354 36771021 PMC9921169

[B53] Kubota Y. Umegaki K. Tanaka N. Mizuno H. Nakamura K. Kunitomo M. (2002). Safety of dietary supplements: chronotropic and inotropic effects on isolated rat atria. Biol. Pharm. Bull. 25 (2), 197–200. 10.1248/bpb.25.197 11853165

[B54] Kumari S. Dhapola R. Sharma P. Nagar P. Medhi B. HariKrishnaReddy D. (2024). The impact of cytokines in neuroinflammation-mediated stroke. Cytokine Growth Factor Rev. 78, 105–119. 10.1016/j.cytogfr.2024.06.002 39004599

[B55] Lai H. F. Huang X. X. Shi Z. L. (2018a). Optimization of ultrasonic-assisted extraction of amentoflavone from *Selaginella uncinate* (*Desv.)spring* using response surface methodology. Food Res. Dev. 39 (13), 47–51+224. 10.3969/j.issn.1005-6521.2018.13.009

[B56] Lai H. F. Pan L. W. Lv G. M. Huang X. X. (2018b). Purification of amentoflavone from *Selaginella uncinate* (*Desv.*)s*pring* by macroporous resin. Jiangsu Agric. Sci. 46 (07), 201–204. 10.15889/j.issn.1002-1302.2018.07.050

[B57] Lee H. Cho S. Kim S. Y. Ju J. Lee S. W. Choi S. (2022). Amentoflavone-enriched *Selaginella rossii* warb. Suppresses body weight and hyperglycemia by inhibiting intestinal lipid absorption in mice fed a high-fat diet. Life (Basel) 12 (4), 472. 10.3390/life12040472 35454963 PMC9024644

[B58] Lee M. M. Cho W. K. Cha M. H. Yim N. H. Yang H. J. Ma J. Y. (2023). The antiviral activity of *Thuja orientalis folium* against Influenza A virus. Virus Res. 335, 199199. 10.1016/j.virusres.2023.199199 37582473 PMC10445455

[B59] Li D. (2020). Protective effect of amentoflavone against myocardial ischemia-reperfusion injury in rats. Zunyi (Guizhou): ZunYi Medical University. dissertation.

[B60] Li W. W. Li D. Qin Y. Sun C. X. Wang Y. L. Gao L. (2021). Cardioprotective effects of Amentoflavone by suppression of apoptosis and inflammation on an *in vitro* and vivo model of myocardial ischemia-reperfusion injury. Int. Immunopharmacol. 101 (Pt B), 108296. 10.1016/j.intimp.2021.108296 34794889

[B61] Li X. (2011). Extraction, characteriazation of Bi-flavonoid from *Selaginella doederleinii* and its interaction with bovine serum albumin. Changsha (Hunan): Central South University. dissertation.

[B62] Li Y. L. Chen X. Niu S. Q. Zhou H. Y. Li Q. S. (2020). Protective antioxidant effects of amentoflavone and total flavonoids from *Hedyotis diffusa* on H_2_O_2_-induced HL-O2 cells through ASK1/p38 MAPK pathway. Chem. Biodivers. 17 (7), e2000251. 10.1002/cbdv.202000251 32342591

[B63] Liao S. Ren Q. Yang C. Zhang T. Li J. Wang X. (2015). Liquid chromatography-tandem mass spectrometry determination and pharmacokinetic analysis of amentoflavone and its conjugated metabolites in rats. J. Agric. Food Chem. 63 (7), 1957–1966. 10.1021/jf5019615 25415840

[B64] Libby P. (2021). Inflammation during the life cycle of the atherosclerotic plaque. Cardiovasc Res. 117 (13), 2525–2536. 10.1093/cvr/cvab303 34550337 PMC8783385

[B65] Li D. Sun C. Yang J. Ma X. Jiang Y. Qiu S. (2019). Ionic liquid-microwave-based extraction of biflavonoids from *Selaginella sinensis* . Molecules 24 (13), 2507. 10.3390/molecules24132507 31324010 PMC6651632

[B66] Liu C. Li W. W. Lei J. Li G. Lu D. M. Xu D. J. (2022). Deep eutectic solvents extraction and optimization of amentoflavone from *Selaginella moellendorffii* . Sci. Technol. Food Ind. 43 (16), 176–184. 10.13386/j.issn1002-0306.2021100160

[B67] Liu T. T. Qian H. Li Y. P. Wu W. W. Min X. W. Yang H. D. (2023). Study on the correlation between lipid accumulation product and risk levels of atherosclerotic cardiovascular disease. Chin. Med. Her. 20 (23), 77–80. 10.20047/j.issn1673-7210.2023.23.16

[B68] Liu Z. Ma H. Sun T. Wang X. Y. Kong M. Z. (2024). Neuroprotective role of amentoflavone on LPS-induced Parkinson's disease animal model via inhibition of microglia-mediated inflammation. J. Biol. Regul. Homeost. Agents 38 (1), 705–718. 10.23812/j.biol.regul.homeost.agents.20243801.58

[B69] Li Y. Y. Lu X. Y. Sun J. L. Wang Q. Q. Zhang Y. D. Zhang J. B. (2019). Potential hepatic and renal toxicity induced by the biflavonoids from *Ginkgo biloba* . Chin. J. Nat. Med. 17 (9), 672–681. 10.1016/S1875-5364(19)30081-0 31526502

[B70] Luo S. (2017). Study on the comprehensive quality control mode and preliminary activity of *selaginella pulvinate* . Hangzhou (Zhejiang): Zhejiang University of Technology. dissertation.

[B71] Ma Y. Q. Lan Y. M. Zhang Z. L. (2021). Research progress of correlation between intestinal microecolofy and cardiovascular and cerebrovasculr disease. J. Northwest Minzu Univ. Nat. Sci. Ed. 42 (04), 21–27. 10.14084/j.cnki.cn62-1188/n.2021.04.007

[B72] Mangmool S. Duangrat R. Rujirayunyong T. Anantachoke N. (2024). Anti-inflammatory effects of the Thai herbal remedy Yataprasen and biflavonoids isolated from *Putranjiva roxburghii* in RAW264.7 macrophages. J. Ethnopharmacol. 327, 117997. 10.1016/j.jep.2024.117997 38442805

[B73] Meng Q. W. Liu H. J. Yi H. R. Liu Q. B. (2024). Mechanisms of NLRP3 inflammasome in atherosclerosis and advances in targeted inflammatory therapy. Zhongguo dong mai ying hua za zhi 32 (01), 79–86. 10.20039/j.cnki.1007-3949.2024.01.011

[B74] Nagai S. Ohara K. Mukai K. (2005). Kinetic study of the quenching reaction of singlet oxygen by flavonoids in ethanol solution. J. Phys. Chem. B 109 (9), 4234–4240. 10.1021/jp0451389 16851486

[B75] Oh J. Rho H. S. Yang Y. Yoon J. Y. Lee J. Hong Y. D. (2013). Extracellular signal-regulated kinase is a direct target of the anti-inflammatory compound amentoflavone derived from *Torreya nucifera* . Mediat. Inflamm. 2013, 761506. 10.1155/2013/761506 PMC3736407 23970815

[B76] Okigawa M. Hwa C. W. Kawano N. Rahman W. (1971). Biflavones in *selaginella* species. Phytochemistry 10, 3286–3287. 10.1016/S0031-9422(00)97392-8

[B77] Oliveira A. R. Jesus P. A. P. Bulhões F. V. Martins Netto E. Oliveira Filho J. Roever L. (2023). Morbimortality and determinants of reperfusion in ischemic stroke. Rev. Assoc. Medica Bras. (1992) 70 (1), e20230472. 10.1590/1806-9282.20230472 PMC10729669 38126448

[B78] Park H. J. Kim M. M. (2019). Amentoflavone induces autophagy and modulates p53. Cell J. 21 (1), 27–34. 10.22074/cellj.2019.5717 30507085 PMC6275431

[B79] Peng Y. Chen Q. Xue Y. H. Jin H. Liu S. Du M. Q. (2024). *Ginkgo biloba* and its chemical components in the management of Alzheimer's disease. Am. J. Chin. Med. 52 (3), 625–666. 10.1142/S0192415X24500277 38654507

[B80] Poznyak A. Grechko A. V. Poggio P. Myasoedova V. A. Alfieri V. Orekhov A. N. (2020). The diabetes mellitus-atherosclerosis connection: the role of lipid and glucose metabolism and chronic inflammation. Int. J. Mol. Sci. 21 (5), 1835. 10.3390/ijms21051835 32155866 PMC7084712

[B81] Qin L. Zhao Y. Zhang B. Li Y. (2018). Amentoflavone improves cardiovascular dysfunction and metabolic abnormalities in high fructose and fat diet-fed rats. Food Funct. 9 (1), 243–252. 10.1039/c7fo01095h 29168869

[B82] Qin T. Huang Z. H. Liu Y. (2021). Research progress of vascular endothelial growth factor and atherosclerotic plaque. Chin. Youjiang Med. J. 49 (06), 465–468. 10.3969/j.issn.1003-1383.2021.06.012

[B83] Qiu F. Zhang L. Zheng J. Cao L. Zhang Z. Deng Y. (2021b). Amentoflavone inhibits M1 polarization of THP-1-derived foam cells by activating PPAR- α/γ. J. South. Med. Univ. 41 (3), 344–351. 10.12122/j.issn.1673-4254.2021.03.05 PMC8075791 33849824

[B84] Qiu S. Zhou Y. Kim J. T. Bao C. Lee H. J. Chen J. (2021a). Amentoflavone inhibits tumor necrosis factor-α-induced migration and invasion through AKT/mTOR/S6k1/hedgehog signaling in human breast cancer. Food Funct. 12 (20), 10196–10209. 10.1039/d1fo01085a 34542136

[B85] Ren Q. X. Wang Y. N. Qu X. Y. Zhen B. Q. Hong T. Zhou Z. (2013a). Preparation and *in vitro* evaluation of self-microemulsifying drug delivery system containing amentoflavone. Chin. J. Exp. Tradit. Med. Formulae 19 (24), 5–9. 10.11653/syfj2013240005

[B86] Ren Q. X. Zhou Z. Wang Q. S. (2013b). Preparation and analytical characterization of micronized amentoflavone by antisolvent freeze-drying method. J. Int. Pharm. Res. 40 (02), 237–241. 10.13220/j.cnki.jipr.2013.02.009

[B87] Ridker P. M. Rifai N. Pfeffer M. Sacks F. Lepage S. Braunwald E. (2000). Elevation of tumor necrosis factor-alpha and increased risk of recurrent coronary events after myocardial infarction. Circulation 101 (18), 2149–2153. 10.1161/01.cir.101.18.2149 10801754

[B88] Rong S. Wan D. Fan Y. Liu S. Sun K. Huo J. (2019). Amentoflavone affects epileptogenesis and exerts neuroprotective effects by inhibiting NLRP3 inflammasome. Front. Pharmacol. 10, 856. 10.3389/fphar.2019.00856 31417409 PMC6682693

[B89] Roth G. A. Mensah G. A. Fuster V. (2020). The global burden of cardiovascular diseases and risks: a compass for global action. J. Am. Coll. Cardiol. 76 (25), 2980–2981. 10.1016/j.jacc.2020.11.021 33309174

[B90] Ruan X. Yan L. Y. Li X. X. Liu B. Zhang H. Wang Q. (2014). Optimization of process parameters of extraction of amentoflavone, quercetin and ginkgetin from *Taxus chinensis* using supercritical-CO2 fluid extraction. Molecules 19 (11), 17682–17696. 10.3390/molecules191117682 25365294 PMC6270813

[B91] Saeedan A. S. Abdel-Rahman R. F. Soliman G. A. Ogaly H. A. Abdel-Kader M. S. (2023). Amentoflavone attenuates oxidative stress and neuroinflammation induced by cerebral ischemia/reperfusion in rats by targeting HMGB1-mediated TLR4/NF-κB signaling pathway. Saudi Pharm. J. 31 (11), 101798. 10.1016/j.jsps.2023.101798 37811125 PMC10551888

[B92] Salaria P. Subrahmanyeswara Rao N. N. Dhameliya T. M. Amarendar Reddy M. (2024). *In silico* investigation of potential phytoconstituents against ligand- and voltage-gated ion channels as antiepileptic agents. 3 Biotech. 14 (4), 99. 10.1007/s13205-024-03948-1 PMC10914661 38456083

[B93] Šamec D. Karalija E. Dahija S. Hassan S. T. S. (2022). Biflavonoids: important contributions to the health benefits of *Ginkgo* (*Ginkgo biloba* L.). Plants (Basel) 11 (10), 1381. 10.3390/plants11101381 35631806 PMC9143338

[B94] Saponara R. Bosisio E. (1998). Inhibition of cAMP-phosphodiesterase by biflavones of *Ginkgo biloba* in rat adipose tissue. J. Nat. Prod. 61 (11), 1386–1387. 10.1021/np970569m 9834158

[B95] Shan C. X. Guo S. C. Yu S. Shan M. Q. Li S. F. Y. Chai C. (2018). Simultaneous determination of quercitrin, afzelin, amentoflavone, hinokiflavone in rat plasma by UFLC-MS-MS and its application to the pharmacokinetics of *Platycladus orientalis* leaves extract. J. Chromatogr. Sci. 56 (10), 895–902. 10.1093/chromsci/bmy066 29982351

[B96] Sharma P. Kishore A. De I. Negi S. Kumar G. Bhardwaj S. (2023). Mitigating neuroinflammation in Parkinson's disease: exploring the role of proinflammatory cytokines and the potential of phytochemicals as natural therapeutics. Neurochem. Int. 170, 105604. 10.1016/j.neuint.2023.105604 37683836

[B97] Shin D. H. Bae Y. C. Kim-Han J. S. Lee J. H. Choi I. Y. Son K. H. (2006). Polyphenol amentoflavone affords neuroprotection against neonatal hypoxic-ischemic brain damage via multiple mechanisms. J. Neurochem. 96 (2), 561–572. 10.1111/j.1471-4159.2005.03582.x 16336627

[B98] Siddiqui A. R. Mushtaq M. Sardar M. Atta L. Nur-E-Alam M. Ahmad A. (2024). Mechanistic insight into the mode of inhibition of dietary flavonoids; targeting macrophage migration inhibitory factor. Front. Mol. Biosci. 11, 1414572. 10.3389/fmolb.2024.1414572 38915940 PMC11194440

[B99] Singh S. Goyal A. Agrawal N. (2023). Molecular docking and dynamic simulation to identify α7nAChR binding affinity of flavonoids for the treatment of alzheimer’s disease. Chem. Biodivers. 20 (7), e202300306. 10.1002/cbdv.202300306 37249245

[B100] Sirimangkalakitti N. Juliawaty L. D. Hakim E. H. Waliana I. Saito N. Koyama K. (2019). Naturally occurring biflavonoids with amyloid β aggregation inhibitory activity for development of anti-Alzheimer agents. Bioorg Med. Chem. Lett. 29 (15), 1994–1997. 10.1016/j.bmcl.2019.05.020 31138471

[B101] Smith E. E. Duchesne S. Gao F. Saad F. Whitehead V. McCreary C. R. (2021). Vascular contributions to neurodegeneration: protocol of the COMPASS-ND study. Can. J. Neurol. Sci. 48 (6), 799–806. 10.1017/cjn.2021.19 33504400

[B102] Sun B. R. Wang Z. Lin Z. Y. (2023). Risk factors of carotid atherosclerosis in elderly patients with type 2 diabetes mellitus complicated with coronary heart disease. Jilin Med. J. 44 (05), 1183–1185.

[B103] Sun L. Sharma A. K. Han B. H. Mirica L. M. (2020). Amentoflavone: a bifunctional metal chelator that controls the formation of neurotoxic soluble Aβ_42_ oligomers. ACS Chem. Neurosci. 11 (17), 2741–2752. 10.1021/acschemneuro.0c00376 32786307 PMC7716246

[B104] Tao Y. Zhu F. Pan M. Liu Q. Wang P. (2022). Pharmacokinetic, metabolism, and metabolomic strategies provide deep insight into the underlying mechanism of *Ginkgo biloba* flavonoids in the treatment of cardiovascular disease. Front. Nutr. 9, 857370. 10.3389/fnut.2022.857370 35399672 PMC8984020

[B105] Tarallo V. Lepore L. Marcellini M. Dal Piaz F. Tudisco L. Ponticelli S. (2011). The biflavonoid amentoflavone inhibits neovascularization preventing the activity of proangiogenic vascular endothelial growth factors. J. Biol. Chem. 286 (22), 19641–19651. 10.1074/jbc.M110.186239 21471210 PMC3103343

[B106] The Writing Committee Of The Report On Cardivascular Health And Diseases Hu S. S. (2023). Report on cardiovascular health and diseases in China 2021: an updated summary. J. Geriatr. Cardiol. 20 (6), 399–430. 10.26599/1671-5411.2023.06.001 37416519 PMC10320777

[B107] Vlasiou M. C. (2023). Cheese and milk adulteration: detection with spectroscopic techniques and HPLC: advantages and disadvantages. Dairy 4 (3), 509–514. 10.3390/dairy4030034

[B108] Wang B. Lu Y. Wang R. Liu S. Hu X. Wang H. (2020a). Transport and metabolic profiling studies of amentoflavone in Caco-2 cells by UHPLC-ESI-MS/MS and UHPLC-ESI-Q-TOF-MS/MS. J. Pharm. Biomed. Anal. 189, 113441. 10.1016/j.jpba.2020.113441 32615340

[B109] Wang G. Li D. Jiang F. Q. Xie X. Y. He Y. Q. (2018b). Optimization of microwave-assisted ionic liquid extraction of amentoflvone from *Selaginella doederleinii* Hieron. Chin. Tradit. Pat. Med. 40 (08), 1851–1855. 10.3969/j.issn.1001-1528.2018.08.040

[B110] Wang G. Tian Y. B. Jiang Y. M. He Q. Yuan S. M. Wei W. (2018c). Optimization of microwave extraction of amentoflavone from *Selaginella doederleinii* by response surface method. Sci. Technol. Food Ind. 39 (07), 146–151. 10.13386/j.issn1002-0306.2018.07.027

[B111] Wang H. (2017). Study on extraction, isolation, characterization and anti-oxidant activities of flavonoids from Podocarpus nagi. Jishou (Hunan): Jishou University. dissertation.

[B112] Wang J. Q. Li M. M. Fan B. Cui W. Y. Wang Q. Lu C. (2023). Research progress on extraction, separation and biological activity of natural Bi-flavonoids. Food Nutr. China, 1–8. 10.19870/j.cnki.11-3716/ts.20231018.001

[B113] Wang L. (2013). Protective effect against hydroxyl radical-induced DNA damage and antioxidant mechanism of amentoflavone. Guangzhou (Guangdong): Guangzhou University of Chinese Medicine. dissertation.

[B114] Wang X. Zhao X. Gu L. Lv C. He B. Liu Z. (2014). Simultaneous determination of five free and total flavonoids in rat plasma by ultra HPLC-MS/MS and its application to a comparative pharmacokinetic study in normal and hyperlipidemic rats. J. Chromatogr. B Anal. Technol. Biomed. Life Sci. 953-954, 1–10. 10.1016/j.jchromb.2014.01.042 24566333

[B115] Wang Y. Chen L. Z. Duan H. T. Wang L. P. Xu C. Ling L. (2018a). Determination of content of amentoflavone in *Selaginella tamariscina* by infrared-assisted extraction method coupled with HPLC. Chin. J. Clin. Pharm. 27 (03), 164–166. 10.19577/j.1007-4406.2018.03.005

[B116] Wang Y. Wang K. Fu J. (2020b). HDAC6 mediates macrophage iNOS expression and excessive nitric oxide production in the blood during endotoxemia. Front. Immunol. 11, 1893. 10.3389/fimmu.2020.01893 32973784 PMC7468378

[B117] Wang Y. Z. Zhang M. Liu Y. Zhao H. L. Zhen X. K. (2015). Pharmacokinetics study on amentoflavone in rats. Chin. Tradit. Pat. Med. 37 (11), 2397–2401. 10.3969/j.issn.1001-1528.2015.11.013

[B118] Wang Z. Wang B. Jin X. (2024). Amentoflavone attenuates homocysteine-induced neuronal ferroptosis-mediated inflammatory response: involvement of the SLC7A11/GPX4 axis activation. Brain Res. Bull. 215, 111005. 10.1016/j.brainresbull.2024.111005 38852649

[B153] Wei L. N. (2010). Study on the separation and purification of amentoflavone from *selaginella tamariscina (beauv.) spring* . Beijing (Beijing): Beijing University of Chemical Technology. dissertation.

[B119] Wei B. Y. Liang M. H. (2021). Research progress of mechanism of diabetes melitus causes atherosclerosis. Chin. J. Cardiovasc Rehabil. Med. 30 (01), 85–87. 10.3969/j.issn.1008-0074.2021.01.22

[B120] Wei D. D. Chen X. Zhao J. J. Hu X. L. Xiong F. Wang H. (2017). Studies on the intestinal absorption kinetics of amentoflavone in rats. Pharm. Clin. Res. 25 (04), 282–285. 10.13664/j.cnki.pcr.2017.04.002

[B121] Wei L. H. Chen T. R. Fang H. B. Jin Q. Zhang S. J. Hou J. (2019). Natural constituents of *St. John's Wort* inhibit the proteolytic activity of human thrombin. Int. J. Biol. Macromol. 134, 622–630. 10.1016/j.ijbiomac.2019.04.181 31047931

[B122] Windsor P. K. Plassmeyer S. P. Mattock D. S. Bradfield J. C. Choi E. Y. Miller B. R. (2021). Biflavonoid-induced disruption of hydrogen bonds leads to amyloid-β disaggregation. Int. J. Mol. Sci. 22 (6), 2888. 10.3390/ijms22062888 33809196 PMC8001082

[B123] Wiszniak S. Schwarz Q. (2021). Exploring the intracrine functions of VEGF-A. Biomolecules 11 (1), 128. 10.3390/biom11010128 33478167 PMC7835749

[B124] Woo E. R. Lee J. Y. Cho I. J. Kim S. G. Kang K. W. (2005). Amentoflavone inhibits the induction of nitric oxide synthase by inhibiting NF-kappaB activation in macrophages. Pharmacol. Res. 51 (6), 539–546. 10.1016/j.phrs.2005.02.002 15829434

[B125] Xi C. C. Gu W. Zhou L. L. (2020). The isolation, identification, antioxidation and anticoagulation activity of *selaginellin* and amentoflavone from *selaginella tamariscina (beauv.) Spring* . Acta Medica Mediterr. 36, 2697–2706. 10.19193/0393-6384_2020_4_414

[B126] Xiao L. Chen Y. Zhang F. Long X. Y. Nie J. Huang Z. J. (2018). Chemical constituents of biflavonoids from *Selaginella uncinata* . J. Pharm. Anal. 38 (12), 2093–2103. 10.16155/j.0254-1793.2018.12.06

[B127] Xiong L. Liu Y. Wang Y. Zhao H. Song X. Fan W. (2024). The protective effect of *Lonicera japonica* Thunb. against lipopolysaccharide-induced acute lung injury in mice: modulation of inflammation, oxidative stress, and ferroptosis. J. Ethnopharmacol. 331, 118333. 10.1016/j.jep.2024.118333 38750986

[B128] Xiong X. Tang N. Lai X. Zhang J. Wen W. Li X. (2021). Insights into amentoflavone: a natural multifunctional biflavonoid. Front. Pharmacol. 12, 768708. 10.3389/fphar.2021.768708 35002708 PMC8727548

[B129] Xu F. Li J. Mao Y. Xu X. J. He J. H. (2013). *Ginkgo biloba* leaf extract research progress. Food Res. Dev. 16, 124–127. 10.3969/j.issn.1005-6521.2013.16.035

[B130] Xu H. Li R. H. Xia Y. S. Yang X. Q. (2021). Research status and prospect of extraction and purification methods of flavonoids. Appl. Chem. Ind. 50 (06), 1677–1682. 10.16581/j.cnki.issn1671-3206.2021.06.010

[B131] Xu J. Wang Y. Zheng T. Huo Y. Du W. (2022). Biflavones inhibit the fibrillation and cytotoxicity of the human islet amyloid polypeptide. J. Mater Chem. B 10 (24), 4650–4661. 10.1039/d2tb00230b 35667301

[B132] Xu Z. Jia S. J. Tan G. S. Li Y. J. (2004). Study on pharmacological activity of biflavones from *Selaginella pulvinata* (hook. Et grev.) Maxim. Chin. J. Mod. Med. 14, 88–89+100.

[B133] Xun L. Yin M. H. (2009). Experiment study on vasodilative effects of amentoflavone ethylacetate extract of *Selaginella tamariscina* . J. Med. Sci. Yanbian Univ. 32 (04), 246–248. 10.16068/j.1000-1824.2009.04.011

[B134] Yan X. D. Guo M. L. (2022). Research progress of the effect of flavonoids on cardiovascular and cerebrovascular ischemic diseases. J. Pharm. Pract. Serv. 40 (02), 97–102. 10.12206/j.issn.1006-0111.202111059

[B135] Yang Y. T. (2023). The effect of EQST on atherosclerosis formation in mice and its mechanism. Guilin (Guangxi): Guangxi Normal University. dissertation.

[B136] Yao W. Li H. Liu Q. Gao Y. Dai J. Bao B. (2016). Cellular metabolomics revealed the cytoprotection of amentoflavone, a natural compound, in lipopolysaccharide-induced injury of human umbilical vein endothelial cells. Int. J. Mol. Sci. 17 (9), 1514. 10.3390/ijms17091514 27618027 PMC5037791

[B137] Yu S. Yan H. Zhang L. Shan M. Chen P. Ding A. (2017). A review on the phytochemistry, pharmacology, and pharmacokinetics of amentoflavone, a naturally-occurring biflavonoid. Molecules 22 (2), 299. 10.3390/molecules22020299 28212342 PMC6155574

[B138] Zhang J. Zhou J. Zhang T. Niu Z. Wang J. Guo J. (2019). Facile fabrication of an amentoflavone-loaded micelle system for oral delivery to improve bioavailability and hypoglycemic effects in KKAy mice. ACS Appl. Mater Interfaces 11 (13), 12904–12913. 10.1021/acsami.9b03275 30860811

[B139] Zhang L. J. (2014). The effects and potential mechanism of total flavonoids of *Selaginella Pulvinata* and amentoflavone against cognitive impairment. Changchun (Jiling): Jiling University. dissertation.

[B140] Zhang Q. Wang Y. L. Gao D. Cai L. Yang Y. Y. Hu Y. J. (2018). Comparing coagulation activity of *Selaginella tamariscina* before and after stir-frying process and determining the possible active constituents based on compositional variation. Pharm. Biol. 56 (1), 67–75. 10.1080/13880209.2017.1421673 29295657 PMC6130545

[B141] Zhang X. Liu Y. Deng G. Huang B. Kai G. Chen K. (2021a). A purified biflavonoid extract from *Selaginella moellendorffii* alleviates gout arthritis via NLRP3/ASC/Caspase-1 Axis suppression. Front. Pharmacol. 12, 676297. 10.3389/fphar.2021.676297 34079466 PMC8165565

[B142] Zhang Z. Sun T. Niu J. G. He Z. Q. Liu Y. Wang F. (2015). Amentoflavone protects hippocampal neurons: anti-inflammatory, antioxidative, and antiapoptotic effects. Neural Regen. Res. 10 (7), 1125–1133. 10.4103/1673-5374.160109 26330838 PMC4541246

[B143] Zhang Z. Wang F. (2013). Progress in research of the biological activity of amentoflavone. Chin. J. New Drugs. 22 (23), 2775–2778.

[B144] Zhang Z. Yang X. Song Y. Q. Tu J. (2021b). Autophagy in Alzheimer's disease pathogenesis: therapeutic potential and future perspectives. Ageing Res. Rev. 72, 101464. 10.1016/j.arr.2021.101464 34551326

[B145] Zhao F. Qian Y. Li H. Yang Y. Wang J. Yu W. (2022). Amentoflavone-loaded nanoparticles enhanced chemotherapy efficacy by inhibition of AKR1B10. Nanotechnology 33 (38), 385101. 10.1088/1361-6528/ac7810 35697009

[B146] Zhao N. Sun C. Zheng M. Liu S. Shi R. (2019). Amentoflavone suppresses amyloid β1-42 neurotoxicity in Alzheimer's disease through the inhibition of pyroptosis. Life Sci. 239, 117043. 10.1016/j.lfs.2019.117043 31722188

[B147] Zheng X. K. Ling T. L. Wang X. L. Liu C. X. Liu Y. Y. Feng W. S. (2011). Effects of total flavonoids and amentoflavone isolated from *Selaginella tamariscina* on human umbilical vein endothelial cells proliferation and VEGF expreesion. Chin. Pharm. J. 46 (13), 998–1002.

[B148] Zheng X. K. Liu C. X. Zhai Y. Y. Li L. L. Wang X. L. Feng W. S. (2013). Protection effect of amentoflavone in *Selaginella tamariscina* against TNF-α-induced vascular injure of endothelial cells. Yao Xue Xue Bao 48 (09), 1503–1509. 10.16438/j.0513-4870.2013.09.014 24358788

[B149] Zheng X. K. Wei Y. Feng W. S. Li Y. J. Zhao X. M. (2008). “Study on hypoglycemic activity of amentoflavone *in vitro* ,” in Communication between biochemistry and molecular biology of traditional Chinese medicine, 182–187.

[B150] Zhou L. N. Wang R. Wang J. J. Zhao K. Zhang T. Hu X. L. (2022). Characterization, solubility and stability of amentoflavone polymorphs. J. Mol. Struct. 1262, 133101. 10.1016/j.molstruc.2022.133101

[B151] Zhou Y. Yang Z. Guo Z. Kang Y. Zhou K. J. (2024). Research progress on the mechanism of *Tianma Gouteng* decoction in preventing and treating nervous system diseases. Glob. Tradit. Chin. Med. 17 (01), 157–165. 10.3969/j.issn.1674-1749.2024.01.031

[B152] Zhuang J. L. (2020). Effects and mechanisms of amentoflavone on lipid accumulation and cell migration. Guangzhou (Guangdong): Guangzhou University of Chinese Medicine. dissertation.

